# Molecular Insights into the Synergistic Effects of Putrescine and Ammonium on Dinoflagellates

**DOI:** 10.3390/ijms25021306

**Published:** 2024-01-21

**Authors:** Yanfei Wang, Kathryn J. Coyne

**Affiliations:** College of Earth, Ocean, and Environment, University of Delaware, Lewes, DE 19958, USA; yfwang@udel.edu

**Keywords:** dinoflagellate, ammonium, putrescine, nitrogen, algicide, polyamine, amine, harmful algae

## Abstract

Ammonium and polyamines are essential nitrogen metabolites in all living organisms. Crosstalk between ammonium and polyamines through their metabolic pathways has been demonstrated in plants and animals, while no research has been directed to explore this relationship in algae or to investigate the underlying molecular mechanisms. Previous research demonstrated that high concentrations of ammonium and putrescine were among the active substances in bacteria-derived algicide targeting dinoflagellates, suggesting that the biochemical inter-connection and/or interaction of these nitrogen compounds play an essential role in controlling these ecologically important algal species. In this research, putrescine, ammonium, or a combination of putrescine and ammonium was added to cultures of three dinoflagellate species to explore their effects. The results demonstrated the dose-dependent and species-specific synergistic effects of putrescine and ammonium on these species. To further explore the molecular mechanisms behind the synergistic effects, transcriptome analysis was conducted on dinoflagellate *Karlodinium veneficum* treated with putrescine or ammonium vs. a combination of putrescine and ammonium. The results suggested that the synergistic effects of putrescine and ammonium disrupted polyamine homeostasis and reduced ammonium tolerance, which may have contributed to the cell death of *K. veneficum*. There was also transcriptomic evidence of damage to chloroplasts and impaired photosynthesis of *K. veneficum*. This research illustrates the molecular mechanisms underlying the synergistic effects of the major nitrogen metabolites, ammonium and putrescine, in dinoflagellates and provides direction for future studies on polyamine biology in algal species.

## 1. Introduction

Polyamines, including putrescine, spermidine, and spermine, among others, are ubiquitous in both eukaryotic and prokaryotic cells [1,2] and are present in all compartments of plant cells [3]. It has been well established that polyamines are essential for plants, animals, and bacteria to regulate cellular processes [4,5,6,7,8]. Modulating intracellular polyamine content is vital for plants to tolerate environmental stresses [3,9,10]. The beneficial effects of polyamines are attributed to their ability to serve as compatible solutes, bind and interact with negatively charged micro- and macromolecules for protection and stabilization, scavenge oxygen and hydroxyl radicals, modulate ion channels, and crosstalk with signal molecules [2,11]. Putrescine is also an obligate precursor for other polyamines during biosynthesis and can be converted sequentially to spermidine and spermine, catalyzed by the spermidine and spermine synthases, respectively [12]. Despite their beneficial effects, high concentrations of polyamines can cause cell damage via the toxic compounds produced during degradation, leading to irreversible reactive oxygen species (ROS) burst and cell death [13]. Polyamines also play a role in initiating programmed cell death (PCD) in plants [10,11] and animals [14]. Less is known about the functions of polyamines in algal species, though research has suggested polyamines in plants and algae may play similar roles [15]. For instance, research has demonstrated the importance of polyamines in growth [16,17], cell division [18], and environmental stress tolerance [19,20,21] in algal species. Polyamines may also participate in the succession of algal blooms [22]. Even less is known about the effects of high concentrations of polyamines on algal species.

The glutamine synthetase/glutamate synthase (GS/GOGAT) pathway is conserved in all living organisms, and glutamate produced by the assimilation of ammonium through this pathway is a precursor for putrescine biosynthesis [23,24,25,26]. Ammonium is often considered the preferred nitrogen source for phytoplankton in the environment due to its lower energy cost for assimilation than nitrate. At high concentrations, however, it can have destructive effects on photosynthetic organisms, resulting in chloroplast damage and photosynthesis disruption, photophosphorylation uncoupling, oxidative stress, and a shift in cellular pH, among many other impacts [27,28]. The flow of nitrogen to polyamines serves as a sink for excess ammonium, indicating an interconnection of these two groups of nitrogen compounds [9,12].

Dinoflagellates are widely distributed in both freshwater and saltwater environments and play significant ecological roles [29]. The photosynthetic dinoflagellates are among the prominent primary producers [30], and some species form symbiotic relationships with reef-building corals [29]. On the other hand, dinoflagellates are known to form persistent and toxic algal blooms that have posed a significant concern for the health of ecosystems, marine animals, and humans [31]. For example, *Karlodinium veneficum*, a representative dinoflagellate species, has caused harmful algal blooms worldwide for decades [32]. This species can produce karlotoxins that are hemolytic, ichthyotoxic, and cytotoxic, which have been associated with massive fish kills [32,33].

Like other algal species, dinoflagellates can utilize ammonium as a nitrogen source [34] but may be more sensitive to ammonium toxicity than other species [28]. Various studies also provide evidence that polyamines play an essential part in the growth and division of these species [16,17]. No research has focused on the addictive and/or synergistic effects of these key nitrogen metabolites on dinoflagellates or any other algal species to date.

Recent research demonstrated putrescine and ammonium were among the active compounds in a dinoflagellate-specific algicide, IRI-160AA, produced by the bacterium *Shewanella* sp. IRI-160 [35,36]. Dinoflagellates exposed to IRI-160AA exhibited several markers for PCD, accompanied by impaired photosynthesis, chloroplast destruction, ROS induction, and cell cycle arrest, among other physiological changes [37,38,39]. The expression of genes related to these physiological changes was also differentially regulated in dinoflagellate *K. veneficum* treated with IRI-160AA [40]. Further metabolomic analysis on *K. veneficum* exposed to IRI-160AA demonstrated altered metabolites involved in ammonium and polyamine metabolism pathways [41]. These results suggested that the inter-connection and/or interaction of these nitrogen compounds may contribute to cell death or, otherwise, play an essential role in these ecologically important algal species.

This research aimed to investigate the synergistic effects of putrescine and ammonium on dinoflagellates, explore the mechanisms behind this effect, and illustrate the interrelationships of these major nitrogen metabolites at the molecular level in these algal species. To achieve this goal, the effects of putrescine with or without added ammonium were evaluated on dinoflagellates *Karlodinium veneficum*, *Prorocentrum minimum*, and *Levanderina fissa* (syn. *Gyrodinium instriatum*). A transcriptome analysis was then conducted for *K. veneficum* treated with putrescine, ammonium, or a combination of putrescine and ammonium to elucidate molecular mechanisms underlying the synergistic effects of putrescine and ammonium on this species.

## 2. Results

### 2.1. Effects of Putrescine with or without Ammonium on Dinoflagellates

**Figure 1:**
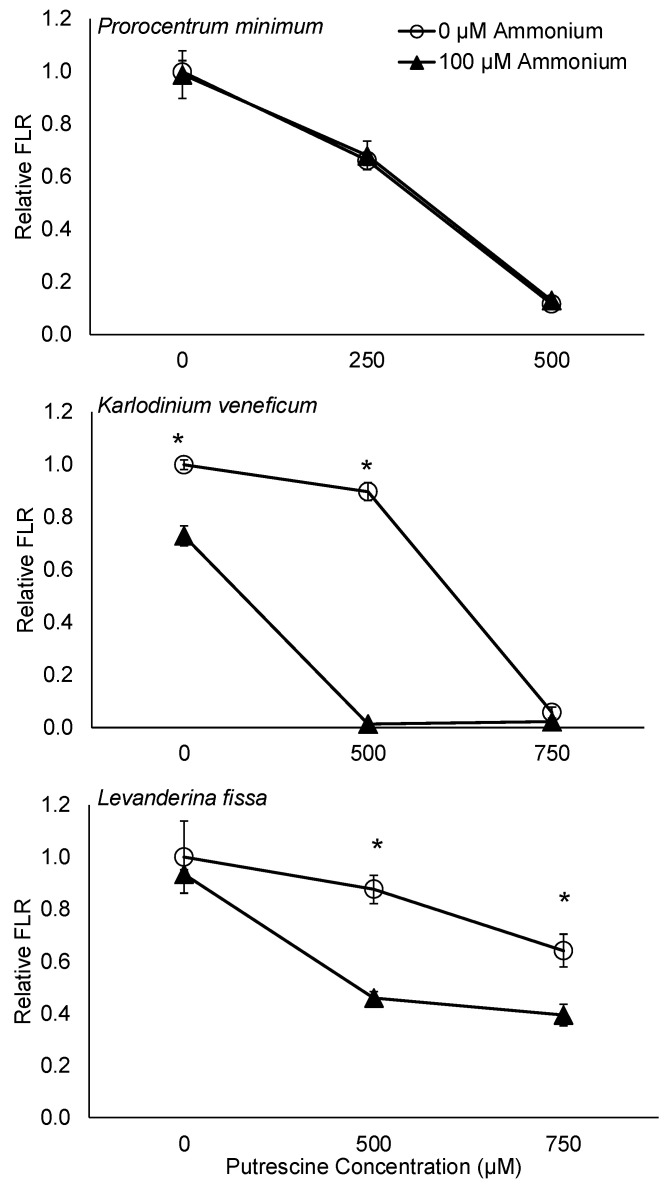
Effects of putrescine with or without ammonium on dinoflagellates. Relative in vivo fluorescence (FLR) of dinoflagellates *Prorocentrum minimum*, *Karlodinium veneficum*, and *Levanderina fissa* treated with different concentrations of putrescine with or without 100 μM ammonium. The bioassay was conducted for 24 h, and the FLR was measured at the end of the experiments. Relative FLR was calculated by dividing the FLR of the treatments by the average FLR of the controls (no ammonium or putrescine added). *K. veneficum* and *L. fissa* were treated with 500 or 750 μM putrescine with or without 100 μM ammonium. *P. minimum* was treated with 250 or 500 μM putrescine with or without 100 μM ammonium. Error bars indicate standard deviations of 3 replicates. Asterisks “*” indicate significant differences in the relative FLR (*p* < 0.05; N = 3) between the 0 µM NH_4_^+^ and the 100 µM NH_4_^+^ treatments.

In vivo fluorescence was used as a proxy for algal biomass as established in other studies [41,42,43,44]. In the ammonium exposure experiments, 100 μM ammonium had a significantly negative effect on the growth of *K. veneficum* (*p* < 0.05) but not on other species tested (*p* > 0.05; Figure 1). The in vivo fluorescence of *K. veneficum* exposed to 100 μM ammonium was 0.73 of the control.

In the putrescine exposure experiments, both 250 and 500 μM putrescine had a significantly negative effect on *P. minimum* (*p* < 0.05; Figure 1), while 500 and 750 μM putrescine had a significantly negative effect on the growth of *K. veneficum* (*p* < 0.05). In vivo fluorescence of *P. minimum* treated with 250 and 500 μM putrescine was 0.66 and 0.12 of the control, respectively; in vivo fluorescence of *K. veneficum* exposed to 500 and 750 μM putrescine was 0.90 and 0.06 of the control, respectively. Exposure to putrescine at 500 μM did not have a significant effect on the growth of *L. fissa* (*p* > 0.05), while 750 μM putrescine had a significantly negative impact on this species (*p* < 0.05). In vivo fluorescence of *L. fissa* treated with 750 μM putrescine was 0.64 of the control.

No significant synergy between putrescine and ammonium was detected on *P. minimum* treated with a combination of 250 or 500 μM putrescine and 100 μM ammonium (*p* > 0.05; Figure 1; Table 1). The relative in vivo fluorescence of *P. minimum* treated with a combination of 250 μM putrescine and 100 μM ammonium was significantly lower compared to the control and the ammonium treatment (*p* < 0.05) but not significantly different from those treated with 250 μM putrescine alone (*p* > 0.05). Similarly, in vivo fluorescence of the treatment exposed to a combination of 500 μM putrescine and 100 μM ammonium was not significantly different from those treated with 500 μM putrescine alone (*p* > 0.05). 

In contrast to *P. minimum*, a significant synergy was detected for *K. veneficum* treated with a combination of 500 μM putrescine and 100 μM ammonium (*p* < 0.05; S_A_S_B_/S_AB_ = 47.08) but not those treated with 750 μM putrescine and 100 μM ammonium (*p* > 0.05; Figure 1; Table 1). Significantly lower relative in vivo fluorescence was observed in the treatment of *K. veneficum* with a combination of 500 μM putrescine and ammonium compared to the control, as well as the treatment with 100 μM ammonium or 500 μM putrescine alone (*p* < 0.05). In addition, the relative in vivo fluorescence of *K. veneficum* treated with a combination of 750 μM putrescine and 100 μM ammonium was significantly lower than the control and the ammonium treatment (*p* < 0.05) but not those treated with 750 μM putrescine (*p* > 0.05). 

A significant synergy was observed for *L. fissa* treated with a combination of 500 μM putrescine and 100 μM ammonium (*p* < 0.05; S_A_S_B_/S_AB_ = 1.78; Figure 1; Table 1), and when treated with a combination of 750 μM putrescine and 100 μM ammonium (*p* < 0.05; S_A_S_B_/S_AB_ = 1.52). A significantly lower relative in vivo fluorescence was observed in *L. fissa* treated with a combination of putrescine (500 or 750 μM) and 100 μM ammonium compared to the respective controls, as well as the respective ammonium and putrescine treatments (*p* < 0.05). 

**Figure 2:**
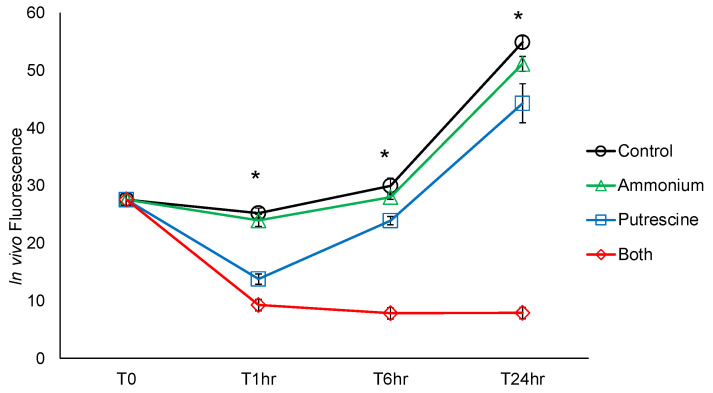
Effects of putrescine with or without ammonium on *Karlodinium veneficum* over time. In vivo fluorescence of *K. veneficum* of the control (no ammonium or putrescine added) and the treatments with 50 μM ammonium, 500 μM putrescine, or a combination of 50 μM ammonium and 500 μM putrescine (the “Both” group). The measurements were taken at the initial time point (T0), 1 (T1hr), 6 (T6hr), and 24 (T24hr) hours after the experiment initiation. Samples for transcriptomic analyses were collected at T1hr. Error bars indicate the standard deviations of 3 replicates. Asterisks “*” indicate significant differences of in vivo fluorescence between groups at the indicated time point (*p* < 0.05; N = 3).

Additionally, due to the sensitivity of *K. veneficum* to 100 μM ammonium, the synergistic effect of ammonium and putrescine was also evaluated by exposing this species to 50 μM ammonium, 500 μM putrescine, or a combination of 50 μM ammonium and 500 μM putrescine (designated as the “Both” treatment). In the entire experiment period of 24 h, no significant difference was observed in the in vivo fluorescence of *K. veneficum* treated with 50 μM ammonium compared to the control (*p* > 0.05; Figure 2). Significantly lower in vivo fluorescence was observed at each of the time points tested (T1hr, T6hr, and T24hr) in the putrescine and Both treatments compared to the control and the ammonium treatment (*p* < 0.05). A significant synergistic effect was observed in *K. veneficum* treated with a combination of 50 μM ammonium and 500 μM putrescine at each of the time points (*p* < 0.05; Table 2). The S_A_S_B_/S_AB_ ratio was 1.41 at T1hr, 2.85 at T6hr, and 5.22 at T24hr. To further investigate the underlying mechanism of the synergistic effect of putrescine and ammonium on *K. veneficum*, samples were collected from this experiment at T1hr for transcriptomic analyses (Section 2.2). 

**Figure 3:**
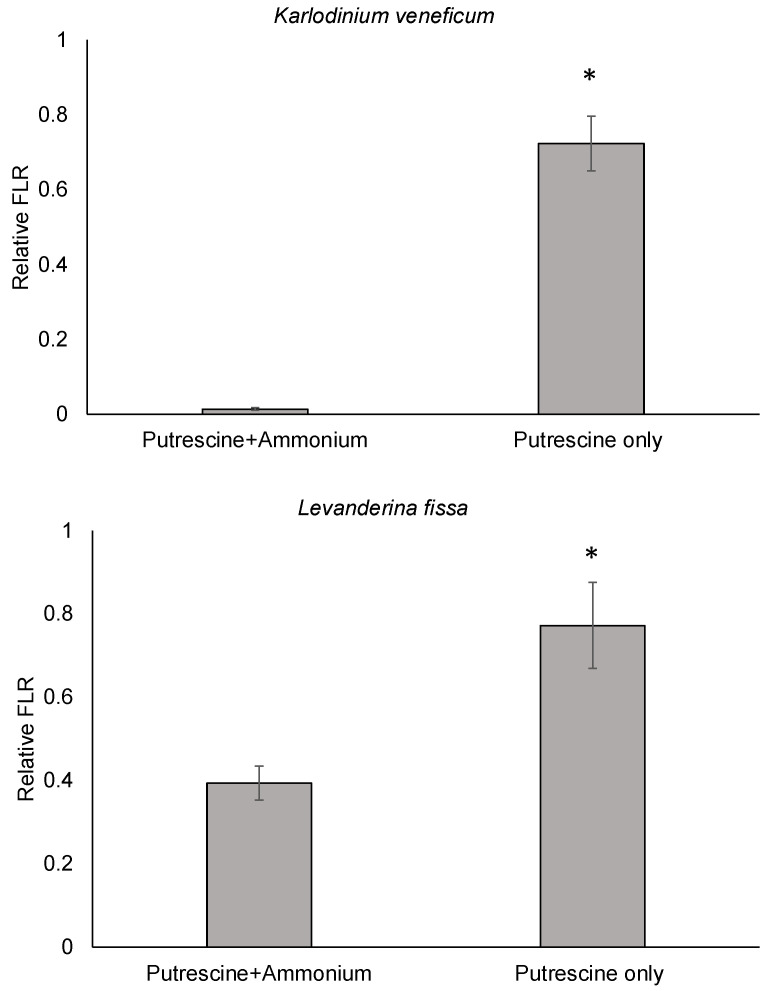
Relative in vivo fluorescence (FLR) of *Karlodinium veneficum* and *Levanderina fissa* exposed to either a combination of putrescine and ammonium or putrescine only, where the amino group (-NH_2_) concentrations were equivalent between treatments of the same species. *K. veneficum* was exposed to 500 μM putrescine plus 100 μM ammonium or 550 μM putrescine only; *L. fissa* was exposed to a combination of 750 μM putrescine and 100 μM ammonium, or 800 μM putrescine only. Control included the same volumes of MilliQ water as in the treatments. The bioassays were conducted for 24 h, and in vivo fluorescence was measured at the end of each bioassay. Relative FLR was calculated by dividing the FLR of the treatments by the average FLR of the controls. Error bars indicate standard deviations of 3 replicates. Asterisks “*” indicate significant differences in the relative FLR (*p* < 0.05; N = 3) between the indicated groups.

Furthermore, to evaluate if the observed synergistic effects of putrescine and ammonium on *K. veneficum* and *L. fissa* were due to an additive effect of amino groups (-NH_2_) in these compounds, these algal species were exposed to either a combination of putrescine (500 and 750 μM for *K. veneficum* and *L. fissa*, respectively) and ammonium (100 μM) or only putrescine (550 and 800 μM for *K. veneficum* and *L. fissa*, respectively), where the -NH_2_ concentrations were equivalent between treatments of the same species (Figure 3). The results indicated a significant difference in the relative in vivo fluorescence of *K. veneficum* and *L. fissa* exposed to a combination of putrescine and ammonium vs. the treatments with only putrescine (*p* < 0.05). The relative in vivo fluorescence of *K. veneficum* exposed to putrescine was 52 times higher compared to the treatment with a combination of putrescine and ammonium. Similarly, the relative in vivo fluorescence of *L. fissa* exposed to putrescine was 1.96 times higher than the treatment with a combination of putrescine and ammonium. 

### 2.2. Transcriptome Analysis of K. veneficum Treated with Ammonium, Putrescine, or a Combination of Ammonium and Putrescine

#### 2.2.1. De Novo Assembly of *K. veneficum* Transcriptome

Transcriptome analysis was performed on *K. veneficum* obtained one hour post exposure. A total of 199,475 transcripts and 164,503 genes were assembled, and 76,454 genes were annotated, accounting for 46.48% of the total assembled genes. A positive and significant correlation between the gene expression from the RT-qPCR and RNA-seq methods was revealed by the gene expression fold-change of the selected genes (R = 0.86, *p* < 0.05; Supplementary File S1, Figure S1). 

**Figure 4:**
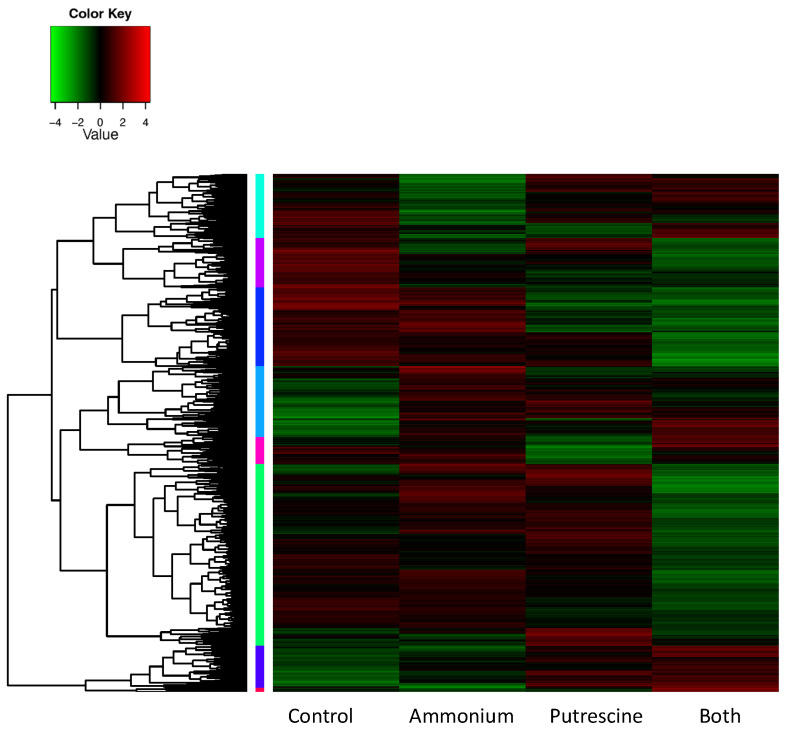
Heatmap of highly differentially expressed genes (DEGs; fold-change > 4, FDR < 0.001). Heatmap of DEGs across the control (no ammonium or putrescine added) and the treatments of *Karlodinium veneficum* amended with ammonium, putrescine, or a combination of ammonium and putrescine (Both). Colors at the left side of the heatmap indicate different sub-groups of genes clustered by their expression patterns.

A total of 25,079 genes were highly differentially expressed (DEGs; FDR < 0.001; fold-change > 4; Supplementary File S2) across the control and treatment, accounting for 15.25% of assembled genes (Figure 4). These DEGs were clustered into eight sub-groups according to their expression patterns. Sequences of all assembled genes are listed in Supplementary File S3.

For pair-wise comparison between each of the treatment and the control, 6306 genes were highly differentially expressed between the ammonium treatment and the control, 7095 between the putrescine treatment and the control, and 15,058 between the Both treatment and the control (Table 3). DEGs in each of the treatments compared to the control accounted for 3.83% of the total assembled genes for the ammonium treatment, 4.31% for the putrescine treatment, and 9.51% for the Both treatment (Table 3). 

#### 2.2.2. Gene Ontology Enrichment Analysis

Gene ontology (GO) enrichment analysis revealed 71 processes were enriched by at least one of the subsets of DEGs (up- or down-regulated by ammonium, putrescine, or a combination of ammonium and putrescine) compared to the control (Supplementary File S1, Figure S2; Table S3). Five processes were enriched by the ammonium up-regulated DEGs and nine by down-regulated DEGs. Up-regulated DEGs in the putrescine treatment were enriched in 6 processes, and down-regulated DEGs were enriched in 16 processes. In addition, 8 processes were enriched by DEGs that were up-regulated in the Both treatment, while 49 processes were enriched by DEGs that were down-regulated in the Both treatment. Among the processes that were enriched by DEGs in the Both treatment, the majority were not shared by DEGs in the ammonium or putrescine treatment; 7 out of 8 (88%) processes enriched by up-regulated DEGs in the Both treatment, and 38 out of 49 (78%) processes enriched by down-regulated DEGs were not shared by the other treatments. 

**Figure 5:**
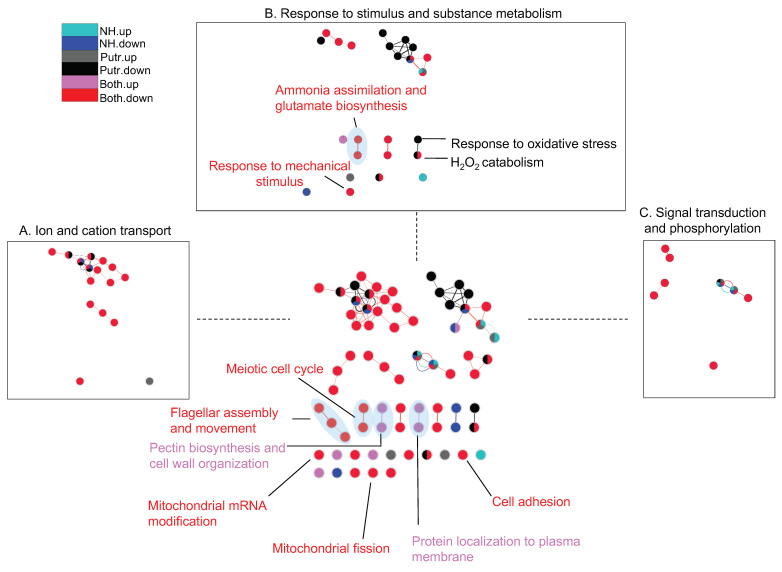
Network analysis of biological processes in *Karlodinium veneficum* enriched by DEGs. Network analysis of biological processes enriched by DEGs up- or down-regulated by ammonium (NH.up and NH.down), putrescine (Putr.up and Putr.down), or a combination of ammonium and putrescine (Both.up and Both.down). Three main groups of processes are highlighted in boxes (A to C; 68% of all processes). Nodes demonstrate biological processes. Edges in the network connect nodes with shared genes, and their thickness is proportional to the overlap between the gene sets represented by the respective nodes [46]. The gene ontology enrichment analysis was conducted using DAVID [47,48]. The network analysis was conducted using the Enrichment Map plugin [46] in Cytoscape software (v 3.8.0) [49].

Forty-eight processes (68% of all enriched biological processes) could be categorized into three groups: ion and cation transport (16 processes; 23% of all enriched biological processes), response to stimulus and substance metabolism (24 processes; 34% of all enriched biological processes), as well as signal transduction and phosphorylation (8 processes; 11% of all enriched biological processes; Figure 5; Supplementary File S1, Tables S3 and S4). 

Nearly all (94%) processes (except for chloride transport) that were related to ion and cation transport were enriched by DEGs that were down-regulated in the Both treatment (Figure 5). Two of these processes (regulation of ion transmembrane transport and regulation of membrane potential) were also enriched by the DEGs that were down-regulated in all treatments. Two processes (sodium ion transmembrane transport and cation transport) were enriched by DEGs that were down-regulated in the Both and putrescine treatments. For processes in this category, the proportion of DEGs in the Both treatment linked to the GO terms relative to the total number of genes associated with the same GO term in the transcriptome (GO-DEG/Total) was 8–37% (Supplementary File S1, Table S4). In comparison, this ratio was 3–4% for DEGs down-regulated by ammonium, and 4–7% for DEGs that were down-regulated by putrescine alone.

Among the 24 processes related to response to stimulus and substance metabolism, 13 (54%) were enriched by DEGs that were down-regulated in the Both treatment (GO-DEG/Total: 15–60%), and 10 (42%) were enriched by DEGs that were down-regulated in the putrescine treatment (GO-DEG/Total: 5–100%) (Figure 5; Supplementary File S1, Tables S3 and S4). One process (cellular response to phosphate starvation) was enriched by DEGs that were down-regulated in all treatments (the ammonium, putrescine, and Both treatments). Two processes (phospholipid catabolic process and hydrogen peroxide catabolic process) were enriched by down-regulated DEGs in the putrescine and Both treatments. Only one process (pectin biosynthetic process) in this category was enriched by DEGs that were up-regulated in the Both treatment (GO-DEG/Total: 30%).

Additionally, all of the eight processes related to signal transduction and phosphorylation were enriched by DEGs that were down-regulated in the Both treatment (GO-DEG/Total: 8–36%; Supplementary File S1, Table S4). Among these processes, protein phosphorylation was also enriched by DEGs that were up- and down-regulated by ammonium and those up-regulated by putrescine. Transmembrane receptor protein tyrosine kinase signaling pathway was also enriched by DEGs that were up- and down-regulated by ammonium as well as DEGs up- and down-regulated by putrescine.

Twenty-three enriched processes (32% of all enriched processes) were not included in the categories above (Figure 5; Supplementary File S1, Tables S3 and S4). Processes related to the meiotic cell cycle, flagellar assembly and movement, and mitochondrial fission were among the processes that were only enriched by down-regulated DEGs in the Both treatment (GO-DEG/Total: 13–75%). 

#### 2.2.3. Genes Involved in Nitrogen Metabolism, Photosynthesis, and Photorepair

**Figure 6:**
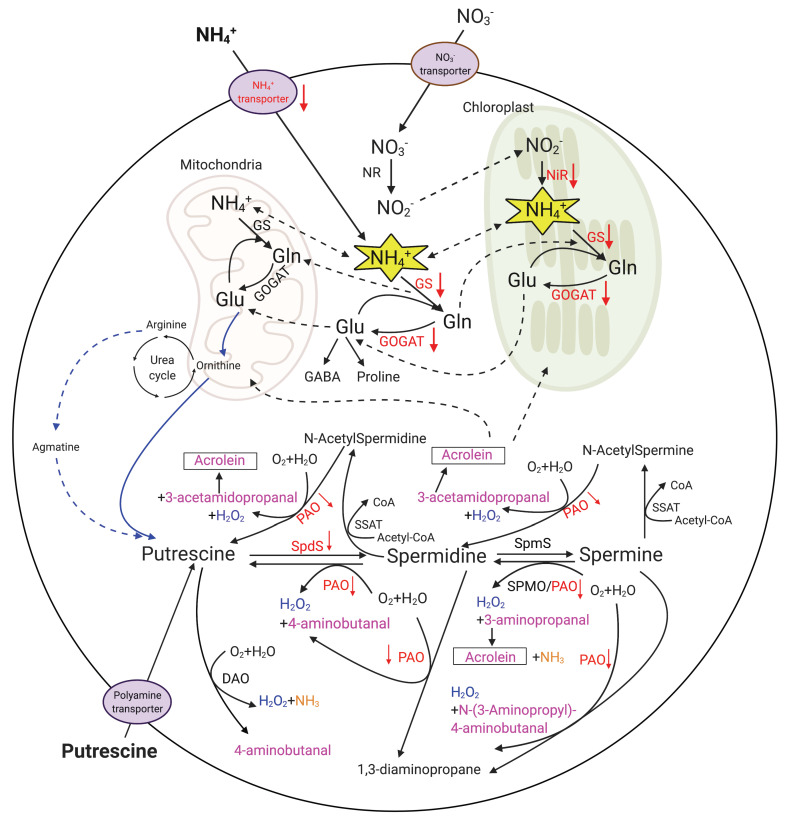
Simplified schematic representation of metabolism of nitrate [50], ammonium [50], and the three major polyamines (putrescine, spermidine, and spermine) in algae [15]. Black solid arrows indicate metabolic pathways; black dashed arrows demonstrate transportation of compounds. Arrows in blue indicate the putrescine biosynthesis pathway from glutamate via an ornithine-urea cycle (OUC). All genes involved in OUC have been identified in dinoflagellates, suggesting these species possess a complete OUC [51]. Also, note that the biosynthesis of putrescine from arginine (dashed blue arrows) may not be universal for all algal species [15]. Enzymes in red are those regulated in *Karlodinium veneficum* in the Both treatment only (treated with a combination of putrescine and ammonium) compared to the control at the transcriptional level revealed by the transcriptome study. Red arrows indicate up- or down-regulation of the genes in the Both treatment compared to the control. The *PAO* (polyamine oxidase) gene identified here is a homolog of the *Arabidopsis AtPAO3*, which is responsible for a “full back-conversion” pathway for polyamine catabolism, including the sequential reaction of spermine to spermidine and spermidine to putrescine, as well as the back-conversion from polyamine N-acetyl-derivatives [52]. Compounds in pink are aldehydes produced during polyamine catabolism. Glu, glutamate; Gln, glutamine; GS, glutamine synthetase; GOGAT, glutamate synthase; NR, nitrate reductase; NiR, nitrite reductase; SpdS, spermidine synthase; SpmS, spermine synthase; SSAT, spermidine/spermine acetyltransferase; SPMO, spermine oxidase; DAO, diamine oxidase. The graph was created with BioRender (BioRender.com; accessed on 17 January 2024).

Five genes encoding ammonium transporters were among the DEGs regulated in the Both treatment (Figure 6; Table 4). All but one (DN158877_c0_g1_i1; *AMT5*) of these genes were down-regulated in the Both treatment compared to the control and were not differentially regulated in the treatment with ammonium or putrescine alone. Furthermore, three *GOGAT* (glutamate synthase; two encoded chloroplastic GOGAT and one encoded a cytosolic GOGAT subunit), a *GS* (glutamine synthetase; cytoplasm), and a *NiR* (nitrite reductase large subunit) were down-regulated in the Both treatment compared to the control and were not differentially regulated by ammonium or putrescine alone. Genes encoding nitrate reductases (NR) were not among the DEGs. Furthermore, a *SpdS* (spermidine synthase) and a *PAO* (polyamine oxidase) were also only down-regulated in the Both treatment compared to the control. 

Genes involved in photosynthesis and photorepair were also differentially regulated in the Both treatment compared to the control (Table 5). The DEGs down-regulated in the Both treatment included *ChlM* (magnesium-protoporphyrin IX methyltransferase), *ChlH* (magnesium-chelatase subunit ChlH), *FCP* (fucoxanthin-chlorophyll a-c binding protein), a gene encoding a pheophorbide a oxygenase, *PP264* (protein LOW PHOTOSYNTHETIC EFFICIENCY 1), *PAPP5* (Serine/threonine-protein phosphatase 5), and *UVR8* (ultraviolet-B receptor UVR8). All of these genes were only down-regulated in the Both treatment and were not differentially regulated in the ammonium or putrescine treatment. Furthermore, two genes, including a *DEGP2* gene (protease Do-like 2) and a *SOD* gene (superoxide dismutase) were up-regulated in the Both treatment compared to the control. The *DEGP2* gene was only up-regulated in the Both treatment, while the *SOD* gene was also up-regulated in the putrescine treatment compared to the control.

## 3. Discussion

Crosstalk between ammonium and polyamines through their metabolic pathways has been reported in animals [56], plants [9,12], and bacteria [8]. So far, no research has explored this relationship in algal species or the molecular mechanism(s) involved. Here, the synergistic effects of ammonium and putrescine were examined for photosynthetic dinoflagellate species *Karlodinium veneficum*, *Prorocentrum minimum*, and *Levanderina fissa*. To further investigate the molecular mechanism(s) behind the observed synergistic effects, transcriptome analysis was conducted on *K. veneficum* treated with ammonium, putrescine, or a combination of putrescine and ammonium (Both).

### 3.1. Synergistic Effects of Putrescine and Ammonium on Dinoflagellates

Dinoflagellates can utilize a wide range of nitrogen sources, yet their growth responses to these sources vary depending on the species. For instance, research indicated *P. minimum* from natural blooms has a preference for ammonium over nitrate, urea, and amino acids, and ammonium was the primary supporting nitrogen in these blooms [57]. Laboratory monoculture experiments demonstrated *P. minimum* and *K. veneficum* had higher intracellular urea pools and greater urease activities compared to other algal species evaluated, suggesting these dinoflagellates may have a competitive advantage in environments with urea as the major nitrogen source [58]. In contrast, a study on *L. fissa* exposed to various inorganic and organic nitrogen sources indicated a better growth of this organism in media with nitrate than ammonium, while it could not utilize organic nitrogen, including urea, for growth [59]. The responses of dinoflagellates to nitrogen sources may also be strain-specific. For instance, a study on dinoflagellate *Gambierdiscus toxicus* strains 1651 and 1655 demonstrated a similar growth rate of strain 1651 cultured with either 50 µM nitrate, ammonium, or putrescine, while strain 1655 had a better growth rate when cultured with ammonium compared to other nitrogen sources [60]. 

In the present study, cultures were maintained in f/2 media with replete nitrogen (882 μM nitrate) [61]. Given these conditions, the non-responsiveness of *K. veneficum* to an added 50 µM ammonium could be expected (Figure 2). Moreover, the absence of adverse effects of ammonium at this concentration on *K. veneficum* suggests that even with the elevated nitrogen content in the treatments, neither nitrate nor ammonium in the media induced direct toxicity to the alga. However, when the ammonium concentration was elevated to 100 μM, a slight but significant reduction was observed in the growth of *K. veneficum* but not the other algal species tested (Figure 1). This indicated a species-dependent response of these organisms to ammonium, which is in line with the studies discussed above. 

It should be noted that natural seawater, which was used in this research, has low inherent nutrient levels, typically below 5 µM for ammonium and less than 15 µM for combined nitrate and nitrite. This low baseline is unlikely to have influenced the experimental results, especially considering the much higher nutrient concentrations employed in this study. For instance, the f/2 medium used for both the control and treatment groups had high nitrate concentrations as mentioned above. Additionally, the treatments included an extra 50 or 100 µM ammonium, with or without putrescine. These substantially higher nutrient levels in the treatments would overshadow the background levels in natural seawater, thus ensuring that the effects observed on algal growth and fluorescence can be attributed to our experimental conditions rather than the ambient nutrient content of the seawater.

In addition, the results of this research demonstrated species- and dose-dependent effects of putrescine on dinoflagellates (Figure 1; Table 1). This was consistent with prior research on the effects of putrescine on both plants [62] and algae [63]. For instance, putrescine stimulated the growth of chlorophyte *Chlorella vulgaris* Beijerinck over a range of 1 to 100 µM, while its growth was inhibited by putrescine at 1 mM [63]. As mentioned above, dinoflagellate *Gambierdiscus toxicus* was also able to grow with 50 µM putrescine as the sole nitrogen source [60]. Nevertheless, no positive effect of putrescine was observed here, even at concentrations that did not have a negative impact (e.g., 500 µM putrescine on *L. fissa*; *p* > 0.05). Preliminary experiments in which *K. veneficum* was exposed to putrescine at low concentrations ranging from 37.5 to 250 µM also showed no significant effect on growth (*p* > 0.05; not published), suggesting that the beneficial effect of putrescine may not be universal or may depend on conditions not examined here. Research on polyamine content in dinoflagellate *Karenia mikimotoi* also demonstrated that the cellular contents of putrescine were not related to the growth rate of this dinoflagellate [16]. This non-universality of putrescine utilization extends to other organisms, such as archaea. In these organisms, a variety of polyamines have been identified, and the sequential process for producing putrescine from arginine and agmatine has been established [64]. However, certain species, such as the halophile *Halobacterium halobium*, are unable to utilize or store external putrescine [64]. 

While the concentrations of putrescine used in this study (250–750 µM) are higher than those typically found in natural environments, the dinoflagellates examined here showed remarkable tolerance to putrescine at elevated levels. For example, *L. fissa* exhibited no significant effects at 500 µM putrescine, while *P. minimum* and *K. veneficum* demonstrated only moderate effects at 250 and 500 µM, respectively (Figure 1). This resilience aligns with findings from other research. A study on the green alga *Chlamydomonas reinhardtii*, for instance, revealed that low putrescine levels (<100 µM) did not affect growth, and higher concentrations (1–10 mM) only slightly reduced their growth rate to 80–90% of the control [65]. These observations, coupled with the growth recovery of *K. veneficum* at 500 µM putrescine (Figure 2), suggest that like other algal species, the dinoflagellates examined here possess a robust pathway for putrescine utilization and catabolism (Figure 6; also see discussion below).

Moreover, the results here indicated that the synergistic effects of ammonium and putrescine were species-specific; a combination of 500 µM putrescine and 100 µM ammonium had the greatest synergistic effect on *K. veneficum* (S_A_S_B_/S_AB_ = 47.08; *p* < 0.05), followed by *L. fissa* (S_A_S_B_/S_AB_ = 1.78; *p* < 0.05), while no significant synergistic effect was observed on *P. minimum* (*p* > 0.05; Table 1). The lack of synergistic effects of ammonium and putrescine on *P. minimum* was consistent with a previous study by Tilney et al. examining the effects of bacterial algicide IRI-160AA on dinoflagellates [39]. IRI-160AA had less effects on the growth of *P. minimum* compared to *K. veneficum* and *L. fissa*. Additionally, IRI-160AA only had negative effects on the photosynthetic parameters of *K. veneficum* and *L. fissa* but not *P. minimum*. These were consistent with a previous report demonstrating a resistance of thecate dinoflagellates (e.g., *P. minimum*) compared to the athecate species (e.g., *K. veneficum* and *L. fissa*) [42]. Tilney et al. also hypothesized that the thecal plates of *P. minimum* may have protected this alga by reducing the plasma membrane accessibility that could be essential for the algicidal activities [39]. Here, it is possible that a similar mechanism was involved, where the thecal plates protected *P. minimum* from the synergistic effects of ammonium and putrescine. The exact mechanism contributing to this resistance requires future research.

Further experiments demonstrated significantly greater (*p* < 0.05) negative effects on *K. veneficum* and *L. fissa* exposed to putrescine plus ammonium vs. putrescine alone with equivalent concentrations of amino groups [-NH_2_] (Figure 3). This indicates that the observed synergistic effects were not due to an additive effect of the introduced ammonium and ammonium/ammonia produced by putrescine oxidation, suggesting the involvement of more complex mechanisms contributing to the synergistic negative effects of ammonium and putrescine. A transcriptomic analysis of *K. veneficum* was then used to investigate the cellular mechanisms underlying these effects (Figure 4). Sample collection for this analysis at one hour after the initial exposure (T1hr) was an appropriate interval due to the rapid turnover of mRNA in algal species responding to external stimuli [66,67,68]. 

### 3.2. Transcriptome Analysis on K. venerficum

The number of genes assembled for *K. veneficum* in this research (164,503) was similar to the number of genes assembled in the same (160,206 genes; [66]) and other dinoflagellates reported previously, including *Cochlodinium polykrikoides* (191,212 genes; [69]) and *Karenia mikimotoi* (202,600 genes; [70]). The low percentage of all DEGs (15.25%), as well as the DEGs in each of the treatments compared to the control (3–9%; Table 3; Figure 4), is also consistent with previous research showing that transcriptional regulation may play a reduced role in dinoflagellates [71]; the protein expression in dinoflagellates is primarily controlled at the post-transcriptional and translational levels [71], and dinoflagellate mRNAs were reported to have substantially longer half-lives than other organisms [72]. However, the wide range of vital biological processes that these DEGs participated in suggests an essential role of transcriptional regulations in these species (Figure 5). Results shown here are similar to previous transcriptomic analyses of *K. veneficum* exposed to bacterial algicide IRI-160AA; while only 3% of all assembled genes were differentially expressed in response to the algicide, they were involved in the regulation of gene expression, protein activity, and morphology, covering various key cellular processes [66]. 

The synergistic effects observed for *K. veneficum* by ammonium and putrescine shown here (Figure 1 and Figure 2) were consistent with transcriptomic analyses, showing that most DEGs were regulated by a combination of these compounds rather than ammonium or putrescine alone (Table 3). This was particularly the case where the highest number of DEGs was observed for those that were down-regulated in the Both treatment (11,215) compared to those up-regulated in the same treatment (3843), or the total DEGs in other treatments (6000–7000) compared to the control. In a similar manner, the majority of biological processes were also down-regulated in the Both treatment compared to treatment with either putrescine or ammonium alone (Figure 5). Furthermore, the identified DEGs involved in nitrogen metabolism illustrated the interconnection between ammonium and putrescine in the Both treatment, suggesting that the crosstalk between these nitrogen species may be vital for the observed synergistic effect (Table 4; Figure 6). The implications of this interconnection are discussed below.

#### 3.2.1. Ammonium May Aggravate Putrescine Toxicity on *K. veneficum*

As an obligate precursor of spermidine and spermine [73], putrescine has a stimulatory effect on the biosynthesis of these polyamines in plants [62] and animals [74]. An increase in polyamines enhances polyamine oxidase (PAO)- and diamine oxidase (DAO)-catalyzed catabolism [13,75], stimulating the production of H_2_O_2_, ammonia, and diverse aldehydes (e.g., 4-aminobutanal, 3-acetamidopropanal, 3-aminopropanal, N-[3-Aminopropyl]-4-aminobutanal, and acrolein; Figure 6) [12,56]. Polyamine accumulation results in the generation of these end-products at toxic levels; the involvement of H_2_O_2_ and aldehydes in polyamine-induced cell damage is well established [2,13,56]. Acrolein, a reactive aldehyde intermediate produced during polyamine oxidation, induces ROS production, membrane damage, and mitochondria dysfunction in animals [76,77]. Elevated ROS production and impaired photosystems were also observed in algal species [78] and cyanobacteria [79] treated with acrolein. In this research, high concentrations of exogenous putrescine likely stimulated the biosynthesis of spermidine and/or spermine, enhancing the catabolic pathways leading to the production of these toxic metabolites (Figure 6). This may have contributed to cell death in the putrescine treatment (Figure 2), while the greater growth inhibition in the Both treatment compared to those treated with putrescine alone suggested that ammonium makes a significant contribution to putrescine-induced toxicity and cell damage. 

To be noted, although polyamines can induce antioxidant enzymes and increase H_2_O_2_ production during their catabolism [80], in this research, the processes related to oxidative stress response and H_2_O_2_ catabolism were enriched by DEGs that were down-regulated in the putrescine and/or Both treatment (Figure 5). This could reflect a disconnect in timing between the collection of samples at T1hr and the more rapid response of the polyamine biosynthesis and catabolism processes and up-regulation of the antioxidant genes [52,81]. For instance, research on *Arabidopsis* PAOs illustrated PAO3 could oxidize over 60% spermidine within 30 min [52], and PAO4 was able to oxidize over 75% of spermine within 15 min [81]. 

The involvement of polyamine oxidation in cell death in the putrescine and Both treatments was also evident by the down-regulated *PAO* (Figure 6; Table 4). The feedback inhibition of plant PAOs by the downstream catabolism products and H_2_O_2_ has been demonstrated [13,52]. A feedback suppression on plant PAOs by ammonium has also been proposed [82], which was consistent with the greater suppressive effect on *PAO* in the Both treatment compared to those treated with putrescine alone. A feedback suppression of *PAO* by the polyamine oxidation products could be a mechanism to alleviate the end-product toxicity [83]. In fact, a partial recovery was observed in the putrescine treatment here (Figure 2), supporting a defense system induced by putrescine, while the observation that there was no recovery in the Both treatment was consistent with the aggravating effect of ammonium in putrescine toxicity. The *PAO* gene identified here is a homolog of the *Arabidopsis AtPAO3*, which is responsible for a “full back-conversion” pathway for polyamine catabolism, including the sequential reaction of spermine to spermidine and spermidine to putrescine, as well as the back-conversion from polyamine N-acetyl-derivatives [52]. The exact role of this regulated PAO enzyme in *K. veneficum* remains unknown. Nevertheless, if this enzyme has a similar function as AtPAO3, the suppression of the *PAO* gene expression in *K. veneficum* may be particularly important in the Both treatment as it can down-regulate diverse downstream processes involved in polyamine catabolism and largely impact polyamine homeostasis.

Similar to *PAO*, *SpdS* encoding a spermidine synthase was also suppressed in the Both treatment and exhibited a trend toward down-regulation in the putrescine treatment, further indicating the feedback suppression of polyamine catabolism products (Figure 6; Table 4). A critical role of SpdS in the homeostatic regulation of polyamines has previously been demonstrated [84,85], and its function in plant survival [86] and development [87], as well as algal growth and cell division [18,85], has been reported. For example, *SpdS*-silenced *Arabidopsis* had reduced spermidine levels, accompanied by abnormal growth and embryo development arrest [84]. Reduced spermidine levels in the green alga *Chlamydomonas reinhardtii* treated with an SpdS inhibitor [85] were accompanied by reduced cell densities [18,85] and cell cycle arrest [18]. Similar to *PAO*, the greater suppression of *SpdS* in the Both treatment compared to those treated with putrescine alone suggested a further polyamine homeostasis disruption with ammonium application, which may play a role in the greater growth inhibition observed in the Both treatment (Figure 2). 

#### 3.2.2. Putrescine May Reduce the Ammonium Tolerance in *K. veneficum*

In addition to the polyamine toxicity and its aggravation by ammonium discussed above, ammonium toxicity likely also played a role in the cell death of *K. veneficum* observed in the Both treatment. Ammonium toxicity occurs when its uptake and cellular production exceed its assimilation [27]; as a primary ammonium assimilation route in both higher plants and algae [25,26], the glutamine synthetase/glutamate synthase (GS/GOGAT) pathway is critical for ammonium detoxification [27,88] (Figure 6). For instance, GS inhibition in diatom *Phaeodactylum tricornutum* resulted in growth inhibition, accompanied by ROS production and chloroplast destruction [89]. It has also been demonstrated that the nitrogen flow through the GS/GOGAT pathway to polyamines can serve as a sink for excess ammonium to reduce its toxicity [9,12]. The disrupted polyamine homeostasis (discussed above) suggests the potential for reduced ammonium tolerance in *K. veneficum* in the Both treatment. This may have been further exacerbated by the reduced ammonium detoxification capacity as evident by the down-regulated *GS* (cytoplasmic) and *GOGAT* (cytosolic and chloroplastic) in the Both treatment (Figure 6; Table 4). 

Furthermore, the down-regulated *NiR* and the GS/GOGAT pathway in the chloroplast may also disrupt the electron flow in this organelle, causing ROS overproduction and cell damage [90,91]. This was consistent with the up-regulation of the *SOD* gene in the Both treatment; *SOD* encodes the key enzyme superoxide dismutase involved in chloroplast oxidative stress reduction and excitation energy dissipation [90] (Table 5). A down-regulation of the ammonium assimilation and glutamate biosynthesis in this treatment was also revealed by the GO enrichment analysis (Figure 5). The greater suppression of the *GS*, *GOGAT*, and *NiR* genes in the Both treatment compared to the putrescine treatment points to a greater impact on the polyamine homeostasis by a combination of putrescine and ammonium compared to putrescine alone. The up-regulation of the *SOD* gene in the putrescine treatment suggests that a high level of putrescine itself can also represent a stressor for the chloroplast redox balance, although it may not be destructive without the addition of ammonium.

A higher intracellular ammonium concentration and/or its toxicity was also supported by the observation that ammonium transporters were repressed only in the Both treatment (Figure 6; Table 4; *AMT1-4*). Transporters of ammonium are sensitive to cellular status and can be turned off when there is an optimal amount of ammonium in the cells, mediated by transcriptional and/or post-transcriptional regulation [27,92]. Research has also demonstrated the suppression of ammonium transporters by GS inhibition in green alga *Ostreococcus tauri* [93] and by glutamine in *Arabidopsis thaliana* [94]. Although trafficking ammonium by transporters normally does not contribute to ammonium toxicity due to the sensitivity of these transporters [82], other pathways (e.g., transportation through aquaporins, non-selective cation channels, K^+^-specific channels, or simple osmotic diffusion) can play a role in transporting an excessive amount of ammonium into the cells [27,82]. These pathways have not been fully characterized, and more research is needed to illustrate their roles in ammonium toxicity in algal species.

Additionally, a previous report identified 68 ammonium transporter domains in dinoflagellate *Symbiodinium microadriaticum*, as well as 42 and 46 in *S. minutum* and *S. kawagutii*, respectively [95]. Among the ammonium transporters in *S. microadriaticum*, only four were differentially expressed in responding to heat stress [95,96]. These genes did not respond to other stressors tested, including cold, salt, and dark [96]. Similar results were revealed in this study, where five DEGs (Table 4) were identified among a total of 56 ammonium transporters, implying the complex nature of ammonium transportation regulation in dinoflagellates. These results were also consistent with the reduced role of gene transcriptional regulation in dinoflagellates, as discussed above [71,72]. Furthermore, the DEGs performing similar roles identified for ammonium transportation and other functions (Table 4 and Table 5) were in line with reports of pervasive gene duplication in dinoflagellates, including genes in tandem arrays [95]. While the mechanisms behind this remain unclear, one hypothesis is that gene duplication in these species could be a strategy to increase transcript abundance and subsequent protein expressions of certain essential genes [95]. Further research should be directed toward understanding the mechanisms behind gene duplication in dinoflagellates and their potential role in the survival and competition of these species in the environment.

#### 3.2.3. A Combination of Putrescine and Ammonium May Damage the Chloroplast and Impair Photosynthesis in *K. veneficum*

Furthermore, the greater impacts of a combination of putrescine and ammonium on the chloroplasts compared to putrescine alone were reflected by the various photosynthesis- and photorepair-related genes that were differentially regulated only in the Both treatment (Table 5). Among these, the only up-regulated gene was *DEGP2* (encoding a protease Do-like 2) involved in the PSII repair [97]. This could reflect an effort to restore the photosynthesis in *K. veneficum* in the Both treatment, while the down-regulation of the other genes implied a limited capacity for recovery. The genes that were down-regulated only in the Both treatment included *FCP* (fucoxanthin-chlorophyll a-c binding protein) encoding the light harvesting complex [98], *PP264* (encoding protein LOW PHOTOSYNTHETIC EFFICIENCY 1) required for light-regulated PSII biogenesis [99], *ChlM* (Magnesium-protoporphyrin IX methyltransferase) [100] and *ChlH* (Magnesium-chelatase subunit ChlH) [101] involved in chlorophyll biosynthesis, as well as a gene encoding pheophorbide a oxygenase involved in chlorophyll degradation [102]. The down-regulation of these genes could be linked to impaired light harvesting complex, PSII, and chlorophyll biosynthesis in the Both treatment, and it implied a lack of ability for *K. veneficum* in the Both treatment to recover from the damage.

#### 3.2.4. A Combination of Putrescine and Ammonium May Inhibit the Growth of *K. veneficum* through the Regulation of Other Essential Processes

In addition to nitrogen metabolism and photosynthesis that may have been affected by the synergistic effects of putrescine and ammonium discussed above, other key processes were also altered by this synergy (Figure 5 and Supplementary File S1, Figure S2, Tables S3 and S4), including mitochondrial fission. Mitochondria play a central role in cells via energy production mediation and by participation in cell cycle regulation and cell death initiation [103]. It has been demonstrated that mitochondria regulate cell death in animals via a mitochondrial outer membrane permeabilization (MOMP) event that triggers the release of soluble proteins, including cytochrome c, Omi, and Smac, leading to caspase activation and apoptosis initiation [104]. The MOMP event can also cause nonapoptotic cell death involving mitochondrial function loss and/or toxic protein release, inducing caspase-independent cell death [104]. Mitochondrial fusion/fission machinery is required for mitochondrial biogenesis, and the fusion/fission balance is essential for maintaining their function while also playing a role in regulating cell cycle progression and cell death [105]. Mitochondrial fission inhibition can cause a hyperfusion in these organelles, leading to genome instability, DNA damage response, and cell cycle arrest in mammalian cells [106]. The down-regulated mitochondrial fission process in the Both treatment suggests mitochondrial dysfunction (Figure 5). Though proteins involved in mitochondrial fission have been identified in other algal species [107], the involvement of this process in regulating algal death and stress response remains unknown and requires future investigation. 

It has been well established that both ammonium [108] and putrescine [109] interact with cation channels and suppress cation transmembrane transport. Here, the diverse down-regulated processes related to ion and cation transport, especially in the Both treatment, were consistent with these roles (Figure 5; Supplementary File S1, Tables S3 and S4). The suppressed ion and cation transport can be especially unfavorable for dinoflagellates, as dinoflagellate DNA contains high concentrations of divalent cations, including Ca^2+^ and Mg^2+^, as well as a large number of transition metals [110], which neutralize the negative charge of naked DNA and allow for its maximal compaction [111]. 

Cations such as Ca^2+^ also play other essential roles in dinoflagellates. For instance, it has been demonstrated that Ca^2+^ is involved in mechanical stimulus response in dinoflagellate *Crypthecodinium cohnii*, and the depletion of Ca^2+^ is involved in the mechanical stimulation-induced cell cycle arrest in this species [112]. Research on dinoflagellate *Cladocopium goreaui* has suggested that ion transport and Ca^2+^ influx may serve as signals and participate in the gene regulation in this species [113]. Ca^2+^ and its signaling also play a role in the flagellar function and cell motility of algal species [114,115,116]. In line with these observations, diverse processes, including mechanical stimulus–response, meiotic cell cycle, flagellar assembly and movement, as well as the majority of the signal transduction pathways, were enriched by the DEGs that were down-regulated in the Both treatment (Figure 5; Supplementary File S1, Table S4). These results implied crosstalk between these essential cellular processes, cation regulation, and the cell growth inhibition induced by the synergistic effects of putrescine and ammonium observed here. 

## 4. Materials and Methods

### 4.1. Algal Stock Cultures

Stock cultures of dinoflagellates *Karlodinium veneficum* (CCMP 2936; National Center for Marine Algae and Microbiota, https://ncma.bigelow.org/; accessed on 17 January 2024), *Prorocentrum minimum* (CCMP2233), and *Levanderina fissa* (CCMP 2935; also known as *Gyrodinium instriatum*) were maintained in natural seawater with f/2 nutrients (-Si; 882 μM nitrate as the sole nitrogen source) [61] and a salinity of 20. The cultures were maintained at 25 °C with a light intensity of approximately 130 µmol photons m^−2^ s^−1^. The cultures were kept semi-continuously in the exponential growth phase under a 12 h:12 h light:dark cycle.

### 4.2. Synergistic Effects of Putrescine and Ammonium on Algal Species

Cultures of *K. veneficum* and *L. fissa* in the f/2 medium (all the f/2 media were made from natural seawater in this research) were amended with 500 or 750 μM putrescine (cat# 51799, MilliporeSigma, Burlington, MA, USA), with 100 μM ammonium (cat# 89503, MilliporeSigma), or with a combination of putrescine and ammonium (N = 3), prepared in MilliQ water. Due to the sensitivity of *P. minimum* to putrescine observed in preliminary experiments, the concentration of putrescine was reduced to 250 and 500 μM putrescine for this species (N = 3). Control cultures only received MilliQ water (N = 3). The bioassay was conducted for 24 h. In vivo fluorescence was used as a reliable proxy for algal biomass in other studies [41,42,43,44]. Here, in vivo fluorescence was measured to track algal growth at the end of each experiment using an AquaFluor handheld fluorometer (Turner, San Jose, CA, USA). The relative in vivo fluorescence was calculated as follows: in vivo fluorescence of each treatment/average in vivo fluorescence of the control [42]. 

In a separate experiment, *K. veneficum* was also treated with either 500 μM putrescine or 50 μM ammonium, or with a combination of 500 μM putrescine and 50 μM ammonium (N = 3; Supplementary File S1, Table S1). Control cultures received MilliQ water and f/2 medium to match the respective volumes added in the treatments (N = 3). Cultures were then incubated for 24 h under the same conditions as the stock cultures. In vivo fluorescence was measured for the bulk culture at the initial time point (T0), and then for each of the control and treatment cultures at 1 h (T1hr), 6 h (T6hr), and 24 h (T24hr) after the initiation of the experiment. Here, the control and treatment were under the light phase at T0, T1hr, and T24hr, while at the dark phase at T6hr.

Synergistic interactions between putrescine and ammonium were evaluated. The relative in vivo fluorescence reflected the surviving fraction (SF) of the cells (also known as proportion of the survived cells) in each of the treatments. Based on Bliss independence [117], the SF of cells under the effect of compounds A (denoted S_A_) and B (denoted S_B_) alone, as well as the SF under the impact of a combination of compounds A and B (denoted S_AB_), would follow the equation S_A_S_B_ = S_AB_ if compounds A and B impact the organisms independently [45]. Synergy between compounds A and B would be indicated by S_A_S_B_ > S_AB_ (or S_A_S_B_/S_AB_ > 1), while S_A_S_B_ < S_AB_ (or S_A_S_B_/S_AB_ < 1) indicates an antagonistic relationship. 

Here, S_A_ was the SF of the ammonium treatment, S_B_ was the SF of the putrescine treatment, and S_AB_ was the SF of the treatment with a combination of ammonium and putrescine. The significance (*p* < 0.05; N = 3) of the synergy was evaluated using a statistical model described previously [45]. If the synergy was significant (*p* < 0.05), then S_A_S_B_/S_AB_ was calculated to reflect how many times more cells would survive in the treatment when adding ammonium and putrescine if the compounds had independent impacts compared to the observed synergistic effects [45]. 

Additionally, to assess if the synergistic effects were due to the combined effects of -NH_2_ in putrescine (2:1—NH_2_:putrescine) and ammonium (1:1), *K. veneficum* was treated with either 500 μM putrescine plus 100 μM ammonium or 550 μM putrescine, where the concentrations of -NH_2_ (1100 μM) were the same in these treatments. A similar experiment was conducted on *L. fissa*, which was treated with either 750 μM putrescine plus 100 μM ammonium or with 800 μM putrescine only, where the -NH_2_ concentrations (1600 μM) were the same between the treatments. All chemicals were dissolved in MilliQ water, and the same volumes of MilliQ water were added to controls without the compounds. The bioassays were conducted for 24 h, and in vivo chlorophyll *a* fluorescence was measured at the end of each bioassay. The relative in vivo chlorophyll *a* fluorescence of each treatment relative to the respective control was calculated as above.

### 4.3. Transcriptome Analysis of K. veneficum Treated with Ammonium, Putrescine, or a Combination of Putrescine and Ammonium

#### 4.3.1. Sample Collection

The samples for transcriptome analysis were collected from *K. veneficum* in the experiment evaluating the synergistic effects of 500 μM putrescine and 50 μM ammonium described above. The samples were collected at T1hr by filtering 40 mL cultures through 3 μm polycarbonate filters (Millipore, Burlington, MA, USA). The filters were immediately immersed in an RLT buffer from an RNeasy Mini Kit (Qiagen, Chatswort, CA, USA) on ice and then stored at −80 °C before RNA extraction, as described below. 

#### 4.3.2. Sample Preparation and RNA-Seq Sequencing

RNA was extracted from filtered cells using the RNeasy Mini Kit (Qiagen) following the manufacturer’s procedure. RNA concentration was determined with a UV-Vis spectrophotometer (NanoDrop Technologies, Wilmington, DE, USA). The integrity of RNA was assessed via electrophoresis on 1% agarose gels. Following the manufacturer’s protocol, contaminating DNA was digested using a DNA-*free*^TM^ DNA Removal Kit (Thermo Fisher Scientific, Waltham, MA, USA). An equal amount of RNA from each replicate collected at T1hr was combined into one composite sample per treatment or control for sequencing. Composite RNA samples were sent to Delaware Biotechnology Institute (DBI; Newark, DE, USA) for library preparation and sequencing. The quantity of the composite RNA was measured using a Qubit fluorometer (Thermo Fisher Scientific). The quality and integrity of the RNA in the composite samples were confirmed using a fragment analyzer (Advanced Analytical Technologies, Inc., Ankeny, IA, USA). 

Messenger RNA (mRNA) was isolated using NEXTflex™ Poly(A) Beads (Perkin-Elmer, Waltham, MA, USA), which limited both ribosomal RNA (rRNA) and prokaryotic mRNA contamination in the final sequence library. A pooled library was prepared using the NEXTFLEX^®^ rapid directional RNA-seq library prep kit (Perkin-Elmer, Waltham, MA, USA) following the manufacturer’s instructions. The library concentration was measured using a Qubit fluorometer (Thermo Fisher Scientific), and the library quality was assessed using a fragment analyzer (Advanced Analytical Technologies, Inc.). Sequencing of 101 base-pair and single-end reads from the pooled library was performed on a single lane using an Illumina HiSeq 2500 system (Illumina, San Diego, CA, USA).

#### 4.3.3. De Novo Assembly and Gene Differential Expression Analysis

Data of the reference genome of *K. veneficum* are not available; de novo assembly of the RNA-seq data was applied here as described previously [66]. Reads from all samples were first assembled into one single assembly using Trinity (v2.8.5) [54,55] with the flag of --trimmomatic to quality trim the reads before the assembly. Bowtie 2 (v2.3.4.3) was used to examine the RNA-seq read representation by the assembly [118]. The contig Nx statistic [55] and ExN50 statistic [119] were estimated using the script provided with the Trinity (v2.8.5) toolkit.

#### 4.3.4. Gene Abundance Estimate and Differential Expression Analysis

Gene abundance in each treatment and control was estimated with Salmon (v0.11.2) [53] bundled with Trinity (v2.8.5) [54,55]. Similar to a previous study [66], downstream analysis was then conducted on all sequences assembled without filtering, as suggested by the Trinity manual (RNA-Seq De novo Assembly Using Trinity; https://github.com/trinityrnaseq/trinityrnaseq/wiki; accessed on 19 October 2023). Differential expression (DE) analysis was conducted at the gene level using edgeR [120], and differentially expressed genes (DEGs; fold-change > 4, FDR < 0.001) were clustered according to their expression patterns using the analyze_diff_expr.pl tool; both programs were bundled in Trinity (v2.8.5) [55]. Additionally, to examine the gene expression patterns, the Trinity toolkit [55] was used to produce Volcano (log(Fold-change) vs. −log10(FDR)) and MA plots (comparing the log-fold-change between experimental groups (M) against the average gene expression across all samples (A); log(Gene Expression) vs. log(Fold-change)).

#### 4.3.5. Gene Annotation

Gene annotation was performed following a previously described protocol [66]. Briefly, TransDecoder (v5.5.0) [55] was used to identify the coding regions by searching against Uniprot/Swiss-prot [121] and protein family (Pfam) [122] databases using BLASTP program (v2.9.0) [123] and HMMER program (v3.2.1) [124], respectively. TransDecoder (v5.5.0) was also used to generate the translated protein sequences [48].

The assembled transcripts from Trinity [54,55] and protein sequences from TransDecoder [55] were then searched against several databases, including the NCBI non-redundant nucleotide (NT) database [125] using the BLASTN program (v2.9.0) [126], the NCBI non-redundant protein sequences (NR) [125] using the BLASTP program [123], and the Uniprot/Swiss-prot database [121] using both BLASTX (v2.9.0) and BLASTP programs [123]. The BLATX program was also used to search against the Eukaryotic Orthologous Groups database (KOG) [127]. All BLAST searches had an e-value cutoff of 0.001. Additionally, the Pfam database [122] was used to annotate the predicted protein sequences with the HMMER program [124]. Finally, the annotation results were loaded into a SQLite database built by the Trinotate program [128]. Kyoto Encyclopedia of Genes and Genomes Ortholog database (KEGG), Gene Ontology (GO), and eggNOG (evolutionary genealogy of genes: Non-supervised Orthologous Groups) [129] terms were assigned via Trinotate [128]. A report was generated using Trinotate with a cut-off *p*-value < 0.001 for all annotations. 

The annotation report was further examined for ribosomal RNA and spliced leader genes to avoid contamination by these genes for further analysis; no such genes were found. Genes that were highly differentially expressed between control and the treatments (fold-change > 4, FDR < 0.001) were further used to construct subsets of annotation reports. Annotations in these subsets were used for further analyses below.

Gene ontology enrichment analysis was performed using DAVID (Database for Annotation, Visualization and Integrated Discovery; v6.8) [47,48] for each subset of data comparing gene regulations between control and the treatments (genes that were up- or down-regulated by ammonium, putrescine, or a combination of ammonium and putrescine compared to the control), following a similar protocol as described previously [66]. Non-repeated Uniprot ID from the above annotation reports was used in this analysis [47,48]. The enriched GO terms were defined as those with a modified Fisher exact *p*-value < 0.05. The annotation of the whole transcriptome was used as the background, and the GO enrichment analysis was conducted using all species in the DAVID database. GO terms in the biological process category at the direct level (only the GO terms directly associated with the genes, not including their parent terms) were included in the following analysis. These GO terms were used to construct a Venn diagram using InteractiVenn [130]. Network analysis was conducted using EnrichmentMap [46] embedded in the Cytoscape software [49] (v 3.8.0). To be noted, while the annotations of sub-clusters of DEGs (Section 4.3.4) were initially examined, GO terms provided a more comprehensive representation of the information in this research. As a result, only GO terms were utilized to summarize gene functions here.

In addition, genes involved in nitrogen metabolism, especially ammonium and polyamine metabolism, as well as genes involved in photosynthesis and photorepair, were searched in the annotation reports generated above. 

#### 4.3.6. Transcriptome Data Validation

The transcriptome data were validated using real-time reverse transcription PCR (RT-qPCR) on selected genes (Supplementary File S1, Table S2. Figure S1). 

### 4.4. Statistical Analysis 

For the bioassays conducted here, one-way ANOVA was used to detect significant differences (*p* < 0.05; N = 3) of the relative in vivo chlorophyll *a* fluorescence between the treatments and the control. If there was a significant difference detected, then the Tukey HSD test was conducted to test the significant differences in the relative in vivo fluorescence between all pairs of groups (*p* < 0.05; N = 3).

## 5. Conclusions

The results of this research revealed a dose-dependent effect of putrescine on dinoflagellates *K. veneficum*, *P. minimum*, and *L. fissa*. Synergistic effects of putrescine and ammonium were observed on *K. veneficum* and *L. fissa* but not on *P. minimum* at the concentrations tested. The results of the transcriptomic analysis on *K. veneficum* suggested the synergistic effects of putrescine and ammonium disrupted polyamine homeostasis, supported by the DEGs involved in polyamine biosynthesis and catabolism. Genes involved in the vital ammonium detoxification route, the GS/GOGAT pathway, were down-regulated in the Both treatment, implying a reduced ammonium toxicity tolerance triggered by the synergistic effects. DEGs involved in chloroplast oxidative stress response and photorepair were also identified in the Both treatment, suggesting damaged chloroplasts and impaired photosynthesis. Furthermore, the enrichment of DEGs in the Both treatment in other key processes, including mitochondrial fission, ion and cation transport, mechanical stimulus response, meiotic cell cycle, flagellar assembly and movement, as well as diverse signal transduction pathways, implied crosstalk between these essential processes and the cell death induced by the synergistic effects. The results of this research illustrate the molecular mechanisms behind the synergistic effects of putrescine and ammonium on dinoflagellates and provide directions for future studies on polyamine biology in algal species.

## Figures and Tables

**Table 1:** Synergistic effects of putrescine and 100 µM ammonium on dinoflagellates *Prorocentrum minimum*, *Karlodinium veneficum*, and *Levanderina fissa*. The significance (*p* < 0.05; N = 3) of synergy was tested using a statistical model described previously [45]. Asterisks “*” indicate the significance of the synergy. S_A_: the surviving fraction (SF) of the ammonium treatments; S_B_: the SF of the putrescine treatments; S_AB_: the SF of those treated with a combination of ammonium and putrescine. S_A_S_B_/S_AB_ was only shown for the groups with a significant synergy.

Species	Putrescine (µM)	Synergy Significance (*p*-Values)	S_A_S_B_/S_AB_
*P. minimum*	250	0.61	/
	500	0.24	/
*K. veneficum*	500	* 3.69 × 10^−7^	47.08
	750	0.16	/
*L. fissa*	500	* 2.03 × 10^−5^	1.78
	750	* 0.0023	1.52

**Table 2:** Synergistic effects of 500 µM putrescine with 50 µM ammonium on dinoflagellate *Karlodinium veneficum* over time. The significance (*p* < 0.05; N = 3) of synergy was tested using the statistical model described previously [45]. Samples at T1hr were collected for transcriptome analysis. Asterisks “*” indicate the significance of the synergy. S_A_: the surviving fraction (SF) of the ammonium treatment; S_B_: the SF of the putrescine treatment; S_AB_: the SF of those treated with a combination of ammonium and putrescine.

Time	Synergy Significance (*p*-Value)	S_A_S_B_/S_AB_
T1hr	* 0.00070	1.41
T6hr	* 2.52 × 10^−7^	2.85
T24hr	* 2.14 × 10^−7^	5.22

**Table 3:** Number of highly differentially expressed genes (DEGs; FDR < 0.001; fold-change > 4) in *Karlodinium veneficum* for each of the treatments compared to the control. Percentage is the percent of total DEGs in each treatment in the 164,503 assembled genes.

Treatment	Up_Regulated	Down-Regulated	Total	Percentage (%)
Ammonium	3562	2744	6306	3.83
Putrescine	3880	3215	7095	4.31
Both	3843	11,215	15,058	9.15

**Table 4:** Expression of highly differentially expressed genes (DEGs; fold-change > 4, FDR < 0.001) involved in ammonium and polyamine metabolism identified in *Karlodinium veneficum* treated with a combination of putrescine and ammonium (Both). Expression of ammonium transporters, GOGAT (glutamate synthase), GOGAT subunit, GS (glutamine synthetase), NiR (nitrite reductase) large subunit, SpdS (spermidine synthase), and PAO (polyamine oxidase) identified in the DEGs of *K. veneficum* treated with a combination of putrescine and ammonium (Both). Con., control; NH_4_, ammonium; Putr., putrescine. Gene expression was measured as TPM (transcripts per million) and normalized using the TMM (trimmed mean of M values) method, generated by Salmon (v0.11.2) [53] embedded in Trinity (v2.8.5) [54,55]. Asterisk “*” indicates the gene was also identified as a DEG in *K. veneficum* treated with ammonium and/or putrescine alone. Sequences are listed in Supplementary File S4.

Transcript ID	Gene Description	Normalized Gene Expression (TMM)			
		Con.	NH_4_	Putr.	Both
DN165880_c0_g1_i1	Ammonium transporter (AMT1)	2.92	2.40	2.67	0.17
DN141316_c0_g1_i1	Ammonium transporter (AMT2)	9.62	7.71	5.08	1.11
DN83039_c0_g1_i1	Ammonium transporter (AMT3)	3.15	5.16	1.08	0.00
DN30808_c0_g1_i1	Ammonium transporter (AMT4)	3.10	0.44	6.82	0.00
DN158877_c0_g1_i1	Ammonium transporter (AMT5)	0.00	* 1.61	* 6.74	3.43
DN149735_c0_g1_i1	NiR large subunit	5.00	4.38	1.81	0.09
DN41374_c0_g1_i3	GOGAT (chloroplast; GOGAT1)	4.11	3.04	1.44	0.28
DN19097_c0_g1_i1	GOGAT (chloroplast; GOGAT2)	4.95	3.51	1.73	0.45
DN100333_c0_g1_i1	GOGAT subunit (cytosol; GOGAT3)	31.30	16.93	8.84	0.00
DN65105_c0_g1_i1	GS (cytoplasm)	4.94	5.73	3.84	0.46
DN111898_c0_g1_i1	SpdS	1.89	2.66	0.28	0.00
DN156058_c0_g1_i1	PAO	3.70	2.50	1.19	0.00

**Table 5:** Expression of highly differentially expressed genes (DEGs; fold-change > 4, FDR < 0.001) involved in photosynthesis and photorepair identified in *Karlodinium veneficum* treated with a combination of putrescine and ammonium (Both). Con., control; NH_4_, ammonium; Putr., putrescine. Gene expression was measured as TPM (transcripts per million) and normalized using the TMM (trimmed mean of M values) method, generated by Salmon (v0.11.2) [53] and embedded in Trinity (v2.8.5) [54,55]. ChlM, magnesium-protoporphyrin IX methyltransferase; FCP, fucoxanthin-chlorophyll a-c binding protein; PP264, protein LOW PHOTOSYNTHETIC EFFICIENCY 1; PAPP5, Serine/threonine-protein phosphatase 5; ChlH, magnesium-chelatase subunit ChlH; DEGP2, protease Do-like 2. SOD, superoxide dismutase. Asterisk “*” indicates the gene was also identified as a DEG in *K. veneficum* treated with putrescine alone. Sequences are listed in Supplementary File S5.

Transcript ID	Gene Description	Normalized Gene Expression (TMM)			
		Con.	NH_4_	Putr.	Both
DN124564_c0_g1_i1	ChlM	1.85	0.46	2.38	0.00
DN154913_c0_g1_i1	FCP	1.89	0.80	2.50	0.00
DN133788_c0_g1_i1	Pheophorbide a oxygenase	3.77	4.95	4.52	0.00
DN101633_c0_g1_i1	PP264 (PP264_1)	7.15	5.02	3.15	0.00
DN152556_c0_g1_i1	PP264 (PP264_2)	3.71	6.08	3.81	0.00
DN97632_c0_g1_i1	PP264 (PP264_3)	2.77	6.00	0.57	0.00
DN68599_c0_g1_i1	PP264 (PP264_4)	14.75	6.70	4.66	1.18
DN99820_c0_g1_i1	PAPP5	3.41	2.76	2.47	0.00
DN128516_c0_g1_i1	UVR8 (UVR8_1)	2.18	4.28	8.94	0.00
DN37485_c0_g1_i1	UVR8 (UVR8_2)	5.31	2.96	1.07	0.45
DN12312_c0_g1_i1	ChlH (ChlH1)	21.42	21.39	10.34	1.02
DN14809_c0_g1_i1	ChlH (ChlH2)	19.58	16.31	10.22	1.15
DN12312_c0_g2_i1	ChlH (ChlH3)	28.43	20.67	15.23	2.58
DN28119_c0_g1_i1	ChlH (ChlH4)	30.15	30.44	14.03	3.78
DN8652_c0_g2_i1	ChlH (ChlH5)	45.86	56.98	22.30	5.04
DN37694_c0_g1_i2	DEGP2	0.72	0.53	2.89	6.38
DN143715_c0_g1_i1	SOD	0.00	0.71	* 3.53	2.45

## Data Availability

The RNA-seq data presented in the study are deposited in the NCBI Sequence Read Archive (SRA; https://www.ncbi.nlm.nih.gov/sra; accessed on 17 January 2024) repository, accession number: SRX19004750, SRX19004751, SRX23258797, SRX23258798.

## Supplementary Materials

The following supporting information can be downloaded at: https://www.mdpi.com/article/10.3390/ijms25021306/s1. Supplementary File S1: Supplementary Methods: RT-qPCR validation of transcriptome data. Supplementary File S2: Detailed information on DEGs of each treatment compared to the control. Supplementary File S3: all sequences generated in this work in the fasta format. Supplementary File S4: sequences of DEGs in Table 4. Supplementary File S5: sequences of DEGs in Table 5. References [131,132] are cited in the supplementary materials.

## References

[1] Sydney T., Sydney T. (2017). Polyamines: Stress Metabolite in Marine Macrophytes.

[2] Nandy S., Das T., Tudu C.K., Mishra T., Ghorai M., Gadekar V.S., Anand U., Kumar M., Behl T., Shaikh N.K. (2022). Unravelling the Multi-Faceted Regulatory Role of Polyamines in Plant Biotechnology, Transgenics and Secondary Metabolomics. Appl. Microbiol. Biotechnol..

[3] Gupta K., Dey A., Gupta B. (2013). Plant Polyamines in Abiotic Stress Responses. Acta Physiol. Plant.

[4] Del Duca S., Serafini-Fracassini D., Cai G. (2014). Senescence and Programmed Cell Death in Plants: Polyamine Action Mediated by Transglutaminase. Front. Plant Sci..

[5] Takahashi T. (2020). Plant Polyamines. Plants.

[6] Navakoudis E., Kotzabasis K. (2022). Polyamines: A Bioenergetic Smart Switch for Plant Protection and Development. J. Plant Physiol..

[7] Miller-Fleming L., Olin-Sandoval V., Campbell K., Ralser M. (2015). Remaining Mysteries of Molecular Biology: The Role of Polyamines in the Cell. J. Mol. Biol..

[8] Krysenko S., Wohlleben W. (2022). Polyamine and Ethanolamine Metabolism in Bacteria as an Important Component of Nitrogen Assimilation for Survival and Pathogenicity. Med. Sci..

[9] Mustafavi S.H., Naghdi Badi H., Sękara A., Mehrafarin A., Janda T., Ghorbanpour M., Rafiee H. (2018). Polyamines and Their Possible Mechanisms Involved in Plant Physiological Processes and Elicitation of Secondary Metabolites. Acta Physiol. Plant.

[10] Pál M., Szalai G., Gondor O.K., Janda T. (2021). Unfinished Story of Polyamines: Role of Conjugation, Transport and Light-Related Regulation in the Polyamine Metabolism in Plants. Plant Sci..

[11] Minocha R., Majumdar R., Minocha S.C. (2014). Polyamines and Abiotic Stress in Plants: A Complex Relationship. Front. Plant Sci..

[12] Moschou P.N., Wu J., Cona A., Tavladoraki P., Angelini R., Roubelakis-Angelakis K.A. (2012). The Polyamines and Their Catabolic Products Are Significant Players in the Turnover of Nitrogenous Molecules in Plants. J. Exp. Bot..

[13] Yu Z., Jia D., Liu T. (2019). Polyamine Oxidases Play Various Roles in Plant Development and Abiotic Stress Tolerance. Plants.

[14] Seiler N., Raul F. (2005). Polyamines and Apoptosis. J. Cell Mol. Med..

[15] Lin H.Y., Lin H.J. (2019). Polyamines in Microalgae: Something Borrowed, Something New. Mar. Drugs.

[16] Nishibori N., Imai I. (2013). Polyamines Control the Growth of the Fish-Killing Dinoflagellate *Karenia mikimotoi* in Culture. Harmful Algae.

[17] Liu Y., Zhao W.H., Dong B., Li B., Yang X.H. (2019). Changes in Intracellular and Extracellular Free Polyamines during the Growth Cycle of Prorocentrum donghaiense. Proceedings of the IOP Conference Series: Earth and Environmental Science, Macao, China, 16–19 July 2019.

[18] Theiss C., Bohley P., Voigt J. (2002). Regulation by Polyamines of Ornithine Decarboxylase Activity and Cell Division in the Unicellular Green Alga *Chlamydomonas reinhardtii*. Plant Physiol..

[19] Sfichi-Duke L., Ioannidis N.E., Kotzabasis K. (2008). Fast and Reversible Response of Thylakoid-Associated Polyamines during and after UV-B Stress: A Comparative Study of the Wild Type and a Mutant Lacking Chlorophyll b of Unicellular Green Alga *Scenedesmus obliquus*. Planta.

[20] Aigner S., Glaser K., Arc E., Holzinger A., Schletter M., Karsten U., Kranner I. (2020). Adaptation to Aquatic and Terrestrial Environments in *Chlorella vulgaris* (Chlorophyta). Front. Microbiol..

[21] Inal M.S., Unal D., Turkyilmaz Unal B., Ozturk M. (2022). Effect of Putrescine on Low-Temperature Acclimation in *Chlamydomonas reinhardtii*. Phyton.

[22] Liu Y., Zhao W., Li C., Miao H. (2017). Free Polyamine Content during Algal Bloom Succession in the East China Sea in Spring 2010. Chin. J. Oceanol. Limnol..

[23] Iwashita Y., Sakiyama T., Musch M.W., Ropeleski M.J., Tsubouchi H., Chang E.B. (2011). Polyamines Mediate Glutamine-Dependent Induction of the Intestinal Epithelial Heat Shock Response. Am. J. Physiol. Gastrointest. Liver Physiol..

[24] Benkerroum N. (2016). Biogenic Amines in Dairy Products: Origin, Incidence, and Control Means. Compr. Rev. Food Sci. Food Saf..

[25] Suárez M.F., Avila C., Gallardo F., Cantón F.R., García-Gutiérrez A., Claros M.G., Cánovas F.M. (2002). Molecular and Enzymatic Analysis of Ammonium Assimilation in Woody Plants. J. Exp. Bot..

[26] Liu N., Li F., Ge F., Tao N., Zhou Q., Wong M. (2015). Mechanisms of Ammonium Assimilation by *Chlorella vulgaris* F1068: Isotope Fractionation and Proteomic Approaches. Bioresour. Technol..

[27] Bittsánszky A., Pilinszky K., Gyulai G., Komives T. (2015). Overcoming Ammonium Toxicity. Plant Sci..

[28] Collos Y., Harrison P.J. (2014). Acclimation and Toxicity of High Ammonium Concentrations to Unicellular Algae. Mar. Pollut. Bull..

[29] Oliveira C.Y.B., Oliveira C.D.L., Müller M.N., Santos E.P., Dantas D.M.M., Gálvez A.O. (2020). A Scientometric Overview of Global Dinoflagellate Research. Publications.

[30] Bi Y., Wang F., Zhang W. (2019). Omics Analysis for Dinoflagellates Biology Research. Microorganisms.

[31] Anderson D.M., Cembella A.D., Hallegraeff G.M. (2012). Progress in Understanding Harmful Algal Blooms: Paradigm Shifts and New Technologies for Research, Monitoring, and Management. Ann. Rev. Mar. Sci..

[32] Place A.R., Bowers H.A., Bachvaroff T.R., Adolf J.E., Deeds J.R., Sheng J. (2012). *Karlodinium veneficum*—The Little Dinoflagellate with a Big Bite. Harmful Algae.

[33] Peng J., Place A., Yoshida W., Anklin C., Hamann M.T. (2008). Structure of Karlotoxin-2, A Toxin Causing Massive Fish Kills Worldwide. Planta Med..

[34] Olofsson M., Robertson E.K., Edler L., Arneborg L., Whitehouse M.J., Ploug H. (2019). Nitrate and Ammonium Fluxes to Diatoms and Dinoflagellates at a Single Cell Level in Mixed Field Communities in the Sea. Sci. Rep..

[35] Ternon E., Wang Y., Coyne K.J. (2019). Small Polar Molecules: A Challenge in Marine Chemical Ecology. Molecules.

[36] Coyne K.J., Wang Y., Johnson G. (2022). Algicidal Bacteria: A Review of Current Knowledge and Applications to Control Harmful Algal Blooms. Front. Microbiol..

[37] Pokrzywinski K.L., Tilney C.L., Modla S., Caplan J.L., Ross J., Warner M.E., Coyne K.J. (2017). Effects of the Bacterial Algicide IRI-160AA on Cellular Morphology of Harmful Dinoflagellates. Harmful Algae.

[38] Pokrzywinski K.L., Tilney C.L., Warner M.E., Coyne K.J. (2017). Cell Cycle Arrest and Biochemical Changes Accompanying Cell Death in Harmful Dinoflagellates Following Exposure to Bacterial Algicide IRI-160AA. Sci. Rep..

[39] Tilney C.L., Pokrzywinski K.L., Coyne K.J., Warner M.E. (2014). Growth, Death, and Photobiology of Dinoflagellates (Dinophyceae) under Bacterial-Algicide Control. J. Appl. Phycol..

[40] Wang Y. (2021). Bacterial Algicides: Application Strategies and Cellular Response of Target Organisms. Ph.D. Dissertation.

[41] Wang Y., Coyne K.J. (2022). Metabolomic Insights of the Effects of Bacterial Algicide IRI-160AA on Dinoflagellate *Karlodinium veneficum*. Metabolites.

[42] Pokrzywinski K.L., Place A.R., Warner M.E., Coyne K.J. (2012). Investigation of the Algicidal Exudate Produced by *Shewanella* sp. IRI-160 and Its Effect on Dinoflagellates. Harmful Algae.

[43] Gustavs L., Schumann R., Eggert A., Karsten U. (2009). In Vivo Growth Fluorometry: Accuracy and Limits of Microalgal Growth Rate Measurements in Ecophysiological Investigations. Aquat. Microb. Ecol..

[44] Wang Y., Coyne K.J. (2020). Immobilization of Algicidal Bacterium *Shewanella* sp. IRI-160 and Its Application to Control Harmful Dinoflagellates. Harmful Algae.

[45] Demidenko E., Miller T.W. (2019). Statistical Determination of Synergy Based on Bliss Definition of Drugs Independence. PLoS ONE.

[46] Merico D., Isserlin R., Stueker O., Emili A., Bader G.D. (2010). Enrichment Map: A Network-Based Method for Gene-Set Enrichment Visualization and Interpretation. PLoS ONE.

[47] Huang D.W., Sherman B.T., Lempicki R.A. (2009). Systematic and Integrative Analysis of Large Gene Lists Using DAVID Bioinformatics Resources. Nat. Protoc..

[48] Huang D.W., Sherman B.T., Lempicki R.A. (2009). Bioinformatics Enrichment Tools: Paths toward the Comprehensive Functional Analysis of Large Gene Lists. Nucleic Acids Res..

[49] Shannon P., Markiel A., Ozier O., Baliga N.S., Wang J.T., Ramage D., Amin N., Schwikowski B., Ideker T. (2003). Cytoscape: A Software Environment for Integrated Models of Biomolecular Interaction Networks. Genome Res..

[50] Glibert P.M., Wilkerson F.P., Dugdale R.C., Raven J.A., Dupont C.L., Leavitt P.R., Parker A.E., Burkholder J.M., Kana T.M. (2016). Pluses and Minuses of Ammonium and Nitrate Uptake and Assimilation by Phytoplankton and Implications for Productivity and Community Composition, with Emphasis on Nitrogen-Enriched Conditions. Limnol. Oceanogr..

[51] Dagenais-Bellefeuille S., Morse D. (2013). Putting the N in Dinoflagellates. Front. Microbiol..

[52] Moschou P.N., Sanmartin M., Andriopoulou A.H., Rojo E., Sanchez-Serrano J.J., Roubelakis-Angelakis K.A. (2008). Bridging the Gap between Plant and Mammalian Polyamine Catabolism: A Novel Peroxisomal Polyamine Oxidase Responsible for a Full Back-Conversion Pathway in *Arabidopsis*. Plant Physiol..

[53] Patro R., Duggal G., Love M., Irizarry R., Kingsford C. (2015). Salmon Provides Accurate, Fast, and Bias-Aware Transcript Expression Estimates Using Dual-Phase Inference. bioRxiv.

[54] Grabherr M.G., Haas B.J., Yassour M., Levin J.Z., Thompson D.A., Amit I., Adiconis X., Fan L., Raychowdhury R., Zeng Q. (2011). Full-Length Transcriptome Assembly from RNA-Seq Data without a Reference Genome. Nat. Biotechnol..

[55] Haas B.J., Papanicolaou A., Yassour M., Grabherr M., Blood P.D., Bowden J., Couger M.B., Eccles D., Li B., Lieber M. (2013). De Novo Transcript Sequence Reconstruction from RNA-Seq Using the Trinity Platform for Reference Generation and Analysis. Nat. Protoc..

[56] Pegg A.E. (2013). Toxicity of Polyamines and Their Metabolic Products. Chem. Res. Toxicol..

[57] Fan C., Glibert P.M., Burkholder J.A.M. (2003). Characterization of the Affinity for Nitrogen, Uptake Kinetics, and Environmental Relationships for *Prorocentrum minimum* in Natural Blooms and Laboratory Cultures. Harmful Algae.

[58] Solomon C.M., Glibert P.M. (2008). Urease Activity in Five Phytoplankton Species. Aquat. Microb. Ecol..

[59] Nagasoe S., Shikata T., Yamasaki Y., Matsubara T., Shimasaki Y., Oshima Y., Honjo T. (2010). Effects of Nutrients on Growth of the Red-Tide Dinoflagellate *Gyrodinium instriatum* Freudenthal et Lee and a Possible Link to Blooms of This Species. Hydrobiologia.

[60] Lartigue J., Jester E.L.E., Dickey R.W., Villareal T.A. (2009). Nitrogen Source Effects on the Growth and Toxicity of Two Strains of the Ciguatera-Causing Dinoflagellate *Gambierdiscus toxicus*. Harmful Algae.

[61] Guillard R.R.L., Ryther J.H. (1962). Studies of Marine Planktonic Diatoms I. *Cyclotella nana* Hustedt, and *Detonula confervasea* (Cleve). Can. J. Microbiol..

[62] Gupta K., Sengupta A., Chakraborty M., Gupta B. (2016). Hydrogen Peroxide and Polyamines Act as Double Edged Swords in Plant Abiotic Stress Responses. Front. Plant Sci..

[63] Czerpak R., Bajguz A., Piotrowska A., Dobrogowska R., Matejczyk M., Wiesławski W. (2003). Biochemical Activity of Di- and Polyamines in the Green Alga *Chlorella vulgaris* Beijerinck (Chlorophyceae). Acta Societatis Botanicorum Poloniae.

[64] Michael A.J. (2018). Polyamine Function in Archaea and Bacteria. J. Biol. Chem..

[65] Freudenberg R.A., Wittemeier L., Einhaus A., Baier T., Kruse O. (2022). Advanced Pathway Engineering for Phototrophic Putrescine Production. Plant Biotechnol. J..

[66] Wang Y., Coyne K.J. (2023). Transcriptome Profiling Reveals a Global Response in Harmful Dinoflagellate *Karlodinium veneficum* to Naturally-Occurring Bacterial Algicides. Front. Mar. Sci..

[67] Wang Y., Bouchard J.N., Coyne K.J. (2018). Expression of Novel Nitrate Reductase Genes in the Harmful Alga, *Chattonella subsalsa*. Sci. Rep..

[68] Sumiya N., Fujiwara T., Kobayashi Y., Misumi O., Miyagishima S.Y. (2014). Development of a Heat-Shock Inducible Gene Expression System in the Red Alga *Cyanidioschyzon merolae*. PLoS ONE.

[69] Guo R., Wang H., Suh Y.S., Ki J.S. (2016). Transcriptomic Profiles Reveal the Genome-Wide Responses of the Harmful Dinoflagellate *Cochlodinium polykrikoides* When Exposed to the Algicide Copper Sulfate. BMC Genom..

[70] Wang X., Niu X., Chen Y., Sun Z., Han A., Lou X., Ge J., Li X., Yang Y., Jian J. (2019). Transcriptome Sequencing of a Toxic Dinoflagellate, *Karenia mikimotoi* Subjected to Stress from Solar Ultraviolet Radiation. Harmful Algae.

[71] Riaz S., Sui Z., Niaz Z., Khan S., Liu Y., Liu H. (2018). Distinctive Nuclear Features of Dinoflagellates with A Particular Focus on Histone and Histone-Replacement Proteins. Microorganisms.

[72] Morey J.S., Van Dolah F.M. (2013). Global Analysis of MRNA Half-Lives and de Novo Transcription in a Dinoflagellate, *Karenia brevis*. PLoS ONE.

[73] Gill S.S., Tuteja N. (2010). Polyamines and Abiotic Stress Tolerance in Plants. Plant Signal Behav..

[74] Neidhart M. (2016). DNA Methylation in Synovial Fibroblasts. DNA Methylation and Complex Human Disease.

[75] Yang J., Wang P., Li S., Liu T., Hu X. (2022). Polyamine Oxidase Triggers H_2_O_2_-Mediated Spermidine Improved Oxidative Stress Tolerance of Tomato Seedlings Subjected to Saline-Alkaline Stress. Int. J. Mol. Sci..

[76] Luo J., Shi R. (2004). Acrolein Induces Axolemmal Disruption, Oxidative Stress, and Mitochondrial Impairment in Spinal Cord Tissue. Neurochem. Int..

[77] Shi R., Rickett T., Sun W. (2011). Acrolein-Mediated Injury in Nervous System Trauma and Diseases. Mol. Nutr. Food Res..

[78] Roach T., Baur T., Stöggl W., Krieger-Liszkay A. (2017). *Chlamydomonas reinhardtii* Responding to High Light: A Role for 2-Propenal (Acrolein). Physiol. Plant.

[79] Shimakawa G., Iwamoto T., Mabuchi T., Saito R., Yamamoto H., Amako K., Sugimoto T., Makino A., Miyake C. (2013). Acrolein, an α,β-Unsaturated Carbonyl, Inhibits Both Growth and PSII Activity in the Cyanobacterium *Synechocystis* sp. PCC 6803. Biosci. Biotechnol. Biochem..

[80] Tanou G., Ziogas V., Belghazi M., Christou A., Filippou P., Job D., Fotopoulos V., Molassiotis A. (2014). Polyamines Reprogram Oxidative and Nitrosative Status and the Proteome of Citrus Plants Exposed to Salinity Stress. Plant Cell Environ..

[81] Fincato P., Moschou P.N., Spedaletti V., Tavazza R., Angelini R., Federico R., Roubelakis-Angelakis K.A., Tavladoraki P. (2011). Functional Diversity inside the *Arabidopsis* Polyamine Oxidase Gene Family. J. Exp. Bot..

[82] Esteban R., Ariz I., Cruz C., Moran J.F., Fernando Moran J. (2016). Review: Mechanisms of Ammonium Toxicity and the Quest for Tolerance. Plant Sei.

[83] Sagor G.H.M., Zhang S., Kojima S., Simm S., Berberich T., Kusano T. (2016). Reducing Cytoplasmic Polyamine Oxidase Activity in *Arabidopsis* Increases Salt and Drought Tolerance by Reducing Reactive Oxygen Species Production and Increasing Defense Gene Expression. Front. Plant Sci..

[84] Imai A., Matsuyama T., Hanzawa Y., Akiyama T., Tamaoki M., Saji H., Shirano Y., Kato T., Hayashi H., Shibata D. (2004). Spermidine Synthase Genes Are Essential for Survival of *Arabidopsis*. Plant Physiol..

[85] Tassoni A., Awad N., Griffiths G. (2018). Effect of Ornithine Decarboxylase and Norspermidine in Modulating Cell Division in the Green Alga *Chlamydomonas reinhardtii*. Plant Physiol. Biochem..

[86] Nambeesan S., Datsenka T., Ferruzzi M.G., Malladi A., Mattoo A.K., Handa A.K. (2010). Overexpression of Yeast Spermidine Synthase Impacts Ripening, Senescence and Decay Symptoms in Tomato. Plant J..

[87] Imamura T., Fujita K., Tasaki K., Higuchi A., Takahashi H. (2015). Characterization of Spermidine Synthase and Spermine Synthase-The Polyamine-Synthetic Enzymes That Induce Early Flowering in Gentiana Triflora. Biochem. Biophys. Res. Commun..

[88] Dayan F.E., Duke S.O. (2014). Natural Compounds as Next-Generation Herbicides. Plant Physiol..

[89] Xie J., Bai X., Li Y., Sun C., Qian H., Fu Z. (2014). The Effect of Glufosinate on Nitrogen Assimilation at the Physiological, Biochemical and Molecular Levels in *Phaeodactylum tricornutum*. Ecotoxicology.

[90] Mullineaux P.M., Exposito-Rodriguez M., Laissue P.P., Smirnoff N. (2018). ROS-Dependent Signalling Pathways in Plants and Algae Exposed to High Light: Comparisons with Other Eukaryotes. Free Radic. Biol. Med..

[91] Karuppanapandian T., Moon J.C., Kim C., Manoharan K., Kim W. (2011). Reactive Oxygen Species in Plants: Their Generation, Signal Transduction, and Scavenging Mechanisms. Aust. J. Crop Sci..

[92] Lanquar V., Frommer W.B. (2010). Adjusting Ammonium Uptake via Phosphorylation. Plant Signal Behav..

[93] Barrios-Llerena M.E., Pritchard J.C., Kerr L.E., Le Bihan T. (2011). The Use of a Novel Quantitation Strategy Based on Reductive Isotopic Di-Ethylation (RIDE) to Evaluate the Effect of Glufosinate on the Unicellular Algae *Ostreococcus tauri*. J. Proteom..

[94] Rawat S.R., Silim S.N., Kronzucker H.J., Siddiqi M.Y., Glass A.D.M. (1999). AtAMT1 Gene Expression and NH_4_^+^ Uptake in Roots of *Arabidopsis thaliana*: Evidence for Regulation by Root Glutamine Levels. Plant J..

[95] Aranda M., Li Y., Liew Y.J., Baumgarten S., Simakov O., Wilson M.C., Piel J., Ashoor H., Bougouffa S., Bajic V.B. (2016). Genomes of Coral Dinoflagellate Symbionts Highlight Evolutionary Adaptations Conducive to a Symbiotic Lifestyle. Sci. Rep..

[96] Baumgarten S., Bayer T., Aranda M., Liew Y.J., Carr A., Micklem G., Voolstra C.R. (2013). Integrating MicroRNA and mRNA Expression Profiling in *Symbiodinium microadriaticum*, a Dinoflagellate Symbiont of Reef-Building Corals. BMC Genom..

[97] Haussühl K., Andersson B., Adamska I. (2001). A Chloroplast DegP2 Protease Performs the Primary Cleavage of the Photodamaged D1 Protein in Plant Photosystem II. EMBO J..

[98] Lang M., Kroth P.G. (2001). Diatom Fucoxanthin Chlorophyll a/c-Binding Protein (FCP) and Land Plant Light-Harvesting Proteins Use a Similar Pathway for Thylakoid Membrane Insertion. J. Biol. Chem..

[99] Jin H., Fu M., Duan Z., Duan S., Li M., Dong X., Liu B., Feng D., Wang J., Peng L. (2018). LOW PHOTOSYNTHETIC EFFICIENCY 1 Is Required for Light-Regulated Photosystem II Biogenesis in *Arabidopsis*. Proc. Natl. Acad. Sci. USA.

[100] Wang Z., Hong X., Hu K., Wang Y., Wang X., Du S., Li Y., Hu D., Cheng K., An B. (2017). Impaired Magnesium Protoporphyrin IX Methyltransferase (ChlM) Impedes Chlorophyll Synthesis and Plant Growth in Rice. Front. Plant Sci..

[101] Adhikari N.D., Froehlich J.E., Strand D.D., Buck S.M., Kramer D.M., Larkin R.M. (2011). GUN4-Porphyrin Complexes Bind the ChlH/GUN5 Subunit of Mg-Chelatase and Promote Chlorophyll Biosynthesis in *Arabidopsis*. Plant Cell.

[102] Xie Q., Liang Y., Zhang J., Zheng H., Dong G., Qian Q., Zuo J. (2016). Involvement of a Putative Bipartite Transit Peptide in Targeting Rice Pheophorbide a Oxygenase into Chloroplasts for Chlorophyll Degradation during Leaf Senescence. J. Genet. Genom..

[103] Tiku V., Tan M.W., Dikic I. (2020). Mitochondrial Functions in Infection and Immunity. Trends Cell Biol..

[104] Tait S.W.G., Green D.R. (2013). Mitochondrial Regulation of Cell Death. Cold Spring Harb. Perspect. Biol..

[105] Horbay R., Bilyy R. (2016). Mitochondrial Dynamics during Cell Cycling. Apoptosis.

[106] Qian W., Choi S., Gibson G.A., Watkins S.C., Bakkenist C.J., Van Houten B. (2012). Mitochondrial Hyperfusion Induced by Loss of the Fission Protein Drp1 Causes ATM-Dependent G2/M Arrest and Aneuploidy through DNA Replication Stress. J. Cell Sci..

[107] Kraus F., Roy K., Pucadyil T.J., Ryan M.T. (2021). Function and Regulation of the Divisome for Mitochondrial Fission. Nature.

[108] Hoopen F.T., Cuin T.A., Pedas P., Hegelund J.N., Shabala S., Schjoerring J.K., Jahn T.P. (2010). Competition between Uptake of Ammonium and Potassium in Barley and *Arabidopsis* Roots: Molecular Mechanisms and Physiological Consequences. J. Exp. Bot..

[109] Pottosin I., Shabala S. (2014). Polyamines Control of Cation Transport across Plant Membranes: Implications for Ion Homeostasis and Abiotic Stress Signaling. Front. Plant Sci..

[110] Gornik S.G., Hu I., Lassadi I., Waller R.F. (2019). The Biochemistry and Evolution of the Dinoflagellate Nucleus. Microorganisms.

[111] Levi-Setti R., Gavrilov K.L., Rizzo P.J. (2008). Divalent Cation Distribution in Dinoflagellate Chromosomes Imaged by High-Resolution Ion Probe Mass Spectrometry. Eur. J. Cell Biol..

[112] Yeung P.K.K., Lam C.M.C., Ma Z.Y., Wong Y.H., Wong J.T.Y. (2006). Involvement of Calcium Mobilization from Caffeine-Sensitive Stores in Mechanically Induced Cell Cycle Arrest in the Dinoflagellate *Crypthecodinium cohnii*. Cell Calcium.

[113] Su Y., Zhang K., Zhou Z., Wang J., Yang X., Tang J., Li H., Lin S. (2020). Microplastic Exposure Represses the Growth of Endosymbiotic Dinoflagellate *Cladocopium goreaui* in Culture through Affecting Its Apoptosis and Metabolism. Chemosphere.

[114] Aiyar P., Schaeme D., García-Altares M., Carrasco Flores D., Dathe H., Hertweck C., Sasso S., Mittag M. (2017). Antagonistic Bacteria Disrupt Calcium Homeostasis and Immobilize Algal Cells. Nat. Commun..

[115] Fort C., Collingridge P., Brownlee C., Wheeler G. (2021). Ca^2+^ Elevations Disrupt Interactions between Intraflagellar Transport and the Flagella Membrane in *Chlamydomonas*. J. Cell Sci..

[116] Kinoshita N., Nagasato C., Motomura T. (2017). Calcium Control of the Sign of Phototaxis in Brown Algal Gametes of *Mutimo cylindricus*. Photochem. Photobiol..

[117] Bliss C.I. (1939). The Toxicity of Poisons Applied Jointly. Ann. Appl. Biol..

[118] Langmead B., Salzberg S.L. (2012). Fast Gapped-Read Alignment with Bowtie 2. Nat. Methods.

[119] Geniza M., Jaiswal P. (2017). Tools for Building de Novo Transcriptome Assembly. Curr. Plant Biol..

[120] Robinson M.D., McCarthy D.J., Smyth G.K. (2009). EdgeR: A Bioconductor Package for Differential Expression Analysis of Digital Gene Expression Data. Bioinformatics.

[121] Bateman A. (2019). UniProt: A Worldwide Hub of Protein Knowledge. Nucleic Acids Res..

[122] Finn R.D., Bateman A., Clements J., Coggill P., Eberhardt R.Y., Eddy S.R., Heger A., Hetherington K., Holm L., Mistry J. (2014). Pfam: The Protein Families Database. Nucleic Acids Res..

[123] Camacho C., Coulouris G., Avagyan V., Ma N., Papadopoulos J., Bealer K., Madden T.L. (2009). BLAST+: Architecture and Applications. BMC Bioinform..

[124] Finn R.D., Clements J., Arndt W., Miller B.L., Wheeler T.J., Schreiber F., Bateman A., Eddy S.R. (2015). HMMER Web Server: 2015 Update. Nucleic Acids Res..

[125] Brown G.R., Hem V., Katz K.S., Ovetsky M., Wallin C., Ermolaeva O., Tolstoy I., Tatusova T., Pruitt K.D., Maglott D.R. (2015). Gene: A Gene-Centered Information Resource at NCBI. Nucleic Acids Res..

[126] Boratyn G.M., Thierry-Mieg J., Thierry-Mieg D., Busby B., Madden T.L. (2019). Magic-BLAST, an Accurate RNA-Seq Aligner for Long and Short Reads. BMC Bioinform..

[127] Tatusov R.L., Fedorova N.D., Jackson J.D., Jacobs A.R., Kiryutin B., Koonin E.V., Krylov D.M., Mazumder R., Smirnov S., Nikolskaya A.N. (2003). The COG Database: An Updated Vesion Includes Eukaryotes. BMC Bioinform..

[128] Bryant D.M., Johnson K., DiTommaso T., Tickle T., Couger M.B., Payzin-Dogru D., Lee T.J., Leigh N.D., Kuo T.H., Davis F.G. (2017). A Tissue-Mapped Axolotl De Novo Transcriptome Enables Identification of Limb Regeneration Factors. Cell Rep..

[129] Huerta-Cepas J., Szklarczyk D., Heller D., Hernández-Plaza A., Forslund S.K., Cook H., Mende D.R., Letunic I., Rattei T., Jensen L.J. (2019). EggNOG 5.0: A Hierarchical, Functionally and Phylogenetically Annotated Orthology Resource Based on 5090 Organisms and 2502 Viruses. Nucleic Acids Res.

[130] Heberle: H., Meirelles V.G., da Silva F.R., Telles G.P., Minghim R. (2015). InteractiVenn: A Web-Based Tool for the Analysis of Sets through Venn Diagrams. BMC Bioinform..

[131] Coyne K.J. (2010). Nitrate reductase (NR1) sequence and expression in the harmful alga *Heterosigma akashiwo* (Raphidophyceae). J. Phycol..

[132] R Core Team (2015). R: A Language and Environment for Statistical Computing.

