# Supplementary material for: Molecular Insights into the Synergistic Effects of Putrescine and Ammonium on Dinoflagellates
Source: Int J Mol Sci. 2024 Jan 21;25(2):1306. doi: 10.3390/ijms25021306 (PMC10816187; doi:10.3390/ijms25021306)
Supplement: Supplementary file 1 [file ijms-25-01306-s001.zip › Supplementary File S1.pdf]

Supplementary Methods. RT-qPCR validation of transcriptome data.

The protocol is similar to the one published by Wang and Coyne (2023). Briefly, the collected samples from the transcriptomic study underwent reverse transcription utilizing the Superscript III First Strand Supermix Kit (Thermo Fisher Scientific Inc., Waltham, MA, USA), adhering to the guidelines provided by the manufacturer. The reactions were primed with random hexamers and terminated at 85 °C for 5 min.

Quantitative real-time PCR (qPCR) was then applied to determine the expression of 7 selected genes plus a reference calmodulin gene (*calm*; DN773_c0_g1) in each of the control and treatment samples. To prepare for the qPCR standards, serial dilutions were made from a cDNA template previously prepared from RNA collected from a *Karlodinium veneficum* stock culture.

Triplicates of 10 μL reactions included 5 μM Power SYBR® Green PCR Master Mix (Thermo Fisher Scientific), 0.9 μM forward primer, 0.9 μM reverse primer, and 1 μl diluted (1:200) template cDNA. Primer sequences are listed in Table S2. The qPCR reactions were conducted on an ABI Prism 7500 Sequence Detection System (Thermo Fisher Scientific). The cycling parameters were 50 °C for 2 min, 95 °C for 10 min, followed by 40 cycles of 95 °C for 15 s, 56 °C for 30 s, and 60 °C for 1 min. A dissociation step was used to evaluate the specificity of the PCR reactions. Standards and negative controls were included in each run. A standard curve was constructed using the Ct values of the standards (Coyne, 2010). The transcript abundance of each gene was determined by linear regression and normalized to the gene expression of the *calm* gene (DN773_c0_g1).

Fold-change of gene expression comparing treatment and control from RT-qPCR (normalized transcript abundance) and transcriptome (expression was measured as TPM [transcripts per million] and normalized using the TMM [trimmed mean of M values] method, generated by Salmon [v0.11.2] (Patro et al., 2015) embedded in Trinity [v2.8.5] (Grabherr et al., 2011; Haas et al., 2013)) was calculated. Only non-zero gene expression was included in the analysis, yielding sixteen RT-qPCR and TMM gene expression fold-change pairs. The correlation between the gene expression fold-change generated from RT-qPCR and RNA-seq sequencing was tested using Pearson's product-moment correlation method with a significance level of 0.05. The statistical analysis was conducted in R (v4.0.2) (R Core Team, 2015).

Table S1. Details of the experimental set-up for the synergistic effects of 50 µM ammonium and 500 µM putrescine on *Karlodinium veneficum*. Transcriptome analysis was done on the samples collected at T1hr of this experiment. Ammonium (50 µM, in sterile MilliQ water), putrescine (500 µM, in sterile MilliQ water), or a combination of 50 µM ammonium and 500 µM putrescine were added to cultures of *K. veneficum* (N=3). The initial culture volume was 98 mL, and the final volume was 100 mL. Control cultures only received sterile MilliQ water and f/2 medium. All treatment and control cultures received the same amount of MilliQ water and f/2 medium.

**Group name**	**Volume of compounds added**
Control	720 µL sterile MilliQ water + 1280 µL f/2 medium
Putrescine	100 µL 500 mM putrescine + 620 µL sterile MilliQ water + 1280 µL f/2 medium
Ammonium	100 µL of 50 mM NH_4Cl stock + 620 µL sterile MilliQ water +1280 µL f/2 medium
Both (putrescine+ ammonium)	100 µL of 500 mM putrescine + 100 µL of 50 mM NH_4Cl + 520 µL sterile MilliQ water + 1280 µL of f/2 medium

Table S2. Primer sequences used in RT-qPCR to validate the transcriptome results. F, forward primers; R, reverse primers. The primers kv_calm_1F/R targeted the reference calmodulin gene (*calm*; DN773_c0_g1) in this study.

Primers	Sequences (5'-3')
kv_calm_1F	GAAGTTGATGCCGACGGTAAT
kv_calm_1R	GCTTCTCGCCTAAGTTTGTCAT
kv_108482_F1	TAAGGGCAGTGTCTTGCGTT
kv_108482_R1	TCGATGTCCCGATGCACAAT
kv_146256_F1	ACCACAAGCATAGTCACCCG
kv_146256_R1	CGCAAAGGTCCCTCTTTCCT
kv_179135_F1	GGAATGGACCGATGGGTGTT
kv_179135_R1	AATGGCTGACCTTGTCCTCG
TRINITY_DN162383_c0_g1_p1F	TGCGTTATTGTTCACTGCCG
TRINITY_DN162383_c0_g1_p1R	CAAGTTCCAAGGGCAAGCTG
TRINITY_DN38745_c0_g1_p1F	AAGGCATTCCAGGAGCTTCG
TRINITY_DN38745_c0_g1_p1R	CCAGACAGGTAGACAGGCAA
TRINITY_DN135495_c0_g1_p1F	GAGCCAATGTTGCGAGGAAG
TRINITY_DN135495_c0_g1_p1R	CCACCTTGCACACCAAGAAC
TRINITY_DN15904_c0_g1_p1F	TTAATGCGTGTGCGTGGTTC
TRINITY_DN15904_c0_g1_p1R	ATCCACATGCTCGTAAGCGT

Table S3. Gene ontology analysis. Detailed gene ontology terms in the biological process category enriched by the DEGs that were up- or down-regulated by ammonium (NH.up/ down), putrescine (Putrescine.up/ down), or a combination of ammonium and putrescine (Both.up/ down) in the transcriptome study. The table shows the detailed Venn diagram analysis result; an overlook of the Venn diagram is shown in Figure S2. The gene ontology analysis was conducted using DAVID (Huang et al., 2009b, 2009a). The Venn diagram analysis was conducted using InteractiVenn (Heberle et al., 2015).

**[NH.up]**
GO:0006952~defense response
**[NH.down]**
GO:1901879~regulation of protein depolymerization
GO:0043171~peptide catabolic process
GO:0042264~peptidyl-aspartic acid hydroxylation
**[Putrescine.up]**
GO:0006821~chloride transport
GO:0030163~protein catabolic process
**[Putrescine.down]**
GO:0071321~cellular response to cGMP
GO:0042493~response to drug
GO:0006979~response to oxidative stress
GO:0009712~catechol-containing compound metabolic process
GO:0050668~positive regulation of homocysteine metabolic process
GO:0045963~negative regulation of dopamine metabolic process
GO:0008203~cholesterol metabolic process
**[Both.up]**
GO:0045489~pectin biosynthetic process
GO:0071555~cell wall organization
GO:0072659~protein localization to plasma membrane
GO:0072661~protein targeting to plasma membrane
GO:0018401~peptidyl-proline hydroxylation to 4-hydroxy-L-proline
GO:0002938~tRNA guanine ribose methylation
GO:0001558~regulation of cell growth
**[Both.down]**
GO:0086010~membrane depolarization during action potential
GO:0071805~potassium ion transmembrane transport
GO:0006814~sodium ion transport
GO:0060285~microtubule-based flagellar cell motility (cilium-dependent cell motility)
GO:0051924~regulation of calcium ion transport

GO:0007155~cell adhesion
GO:0006584~catecholamine metabolic process
GO:0070588~calcium ion transmembrane transport
GO:0000160~phosphorelay signal transduction system
GO:0007186~G-protein coupled receptor signaling pathway
GO:0055085~transmembrane transport
GO:0051291~protein heterooligomerization
GO:0009612~response to mechanical stimulus
GO:0072718~response to cisplatin
GO:0007166~cell surface receptor signaling pathway
GO:0070192~chromosome organization involved in meiotic cell cycle
GO:0080156~mitochondrial mRNA modification
GO:0072384~organelle transport along microtubule
GO:0006874~cellular calcium ion homeostasis
GO:0003341~flagellar movement (cilium movement)
GO:0006813~potassium ion transport
GO:1903351~cellular response to dopamine
GO:0090263~positive regulation of canonical Wnt signaling pathway
GO:0007131~reciprocal meiotic recombination
GO:0030705~cytoskeleton-dependent intracellular transport
GO:0019676~ammonia assimilation cycle
GO:0033132~negative regulation of glucokinase activity
GO:0055074~calcium ion homeostasis
GO:0098719~sodium ion import across plasma membrane
GO:0023014~signal transduction by protein phosphorylation
GO:0055075~potassium ion homeostasis
GO:0018107~peptidyl-threonine phosphorylation
GO:0090526~regulation of gluconeogenesis involved in cellular glucose homeostasis
GO:0055091~phospholipid homeostasis
GO:0097054~L-glutamate biosynthetic process
GO:0071320~cellular response to cAMP
GO:0000266~mitochondrial fission
GO:0036159~inner dynein arm assembly
**[NH.up] and [Putrescine.up]**
GO:0043059~regulation of forward locomotion
**[NH.down] and [Both.up]**
GO:0032259~methylation
**[Putrescine.down] and [Both.down]**

GO:0007018~microtubule-based movement
GO:0042744~hydrogen peroxide catabolic process
GO:0035725~sodium ion transmembrane transport
GO:0009395~phospholipid catabolic process
GO:0006812~cation transport
**[NH.down] and [Putrescine.down] and [Both.down]**
GO:0016036~cellular response to phosphate starvation
GO:0034765~regulation of ion transmembrane transport
GO:0042391~regulation of membrane potential
**[NH.up] and [NH.down] and [Putrescine.up] and [Both.down]**
GO:0006468~protein phosphorylation
**[NH.up] and [Putrescine.up] and [Both.down]**
GO:0042420~dopamine catabolic process
**[NH.up] and [NH.down] and [Putrescine.up] and [Putrescine.down] and [Both.down]**
GO:0007169~transmembrane receptor protein tyrosine kinase signaling pathway

Table S4. Enriched biological processes related to ion and cation transport, response to stimulus and organic substance metabolism, signal transduction and phosphorylation, and other processes in the transcriptome analysis. NH.up/down: DEGs that were up- or down-regulated in the ammonium treatment; Putrescine.up/down: DEGs that were up- or down-regulated in the putrescine treatment; Both.up/down: DEGs that were up- or down-regulated in the treatment with a combination of putrescine and ammonium. The gene ontology analysis was conducted using DAVID (Huang et al., 2009b, 2009a). Numbers in parentheses "()" represent the proportion of DEGs pertinent to the respective GO term within each dataset (NH.up/down, Putrescine.up/down, and Both.up/down) relative to the total number of genes associated with that GO term in the transcriptome (GO-DEG/Total).

Processes	Enriched DEG subset(s)
**Ion and cation transport**	
GO:0086010~membrane depolarization during action potential	[Both.down] (25%)
GO:0071805~potassium ion transmembrane transport	[Both.down] (14%)
GO:0006814~sodium ion transport	[Both.down] (16%)
GO:0051924~regulation of calcium ion transport	[Both.down] (37%)
GO:0070588~calcium ion transmembrane transport	[Both.down] (13%)
GO:0006874~cellular calcium ion homeostasis	[Both.down] (12%)
GO:0006813~potassium ion transport	[Both.down] (10%)
GO:0055074~calcium ion homeostasis	[Both.down] (27%)
GO:0098719~sodium ion import across plasma membrane	[Both.down] (27%)
GO:0055075~potassium ion homeostasis	[Both.down] (25%)
GO:0055085~transmembrane transport	[Both.down] (8%)
GO:0034765~regulation of ion transmembrane transport	[NH.down] (3%)
	[Putrescine.down] (4%)
	[Both.down] (18%)
GO:0042391~regulation of membrane potential	[NH.down] (4%)
	[Putrescine.down] (4%)
	[Both.down] (19%)
GO:0035725~sodium ion transmembrane transport	[Putrescine.down] (7%)
	[Both.down] (21%)
GO:0006812~cation transport	[Putrescine.down] (6%)
	[Both.down] (11%)
GO:0006821~chloride transport	[Putrescine.up] (9%)
**Response to stimulus and organic substance metabolism**	
GO:0072718~response to cisplatin	[Both.down] (44%)
GO:1903351~cellular response to dopamine	[Both.down] (60%)
GO:0071320~cellular response to cAMP	[Both.down] (15%)
GO:0006584~catecholamine metabolic process	[Both.down] (35%)
GO:0009612~response to mechanical stimulus	[Both.down] (23%)

GO:0097054~L-glutamate biosynthetic process	[Both.down] (43%)
GO:0033132~negative regulation of glucokinase activity	[Both.down] (50%)
GO:0090526~regulation of gluconeogenesis involved in cellular glucose homeostasis	[Both.down] (43%)
GO:0019676~ammonia assimilation cycle	[Both.down] (50%)
GO:0045489~pectin biosynthetic process	[Both.up] (30%)
GO:0043171~peptide catabolic process	[NH.down] (8%)
GO:0016036~cellular response to phosphate starvation	[NH.down] (14%)
	[Putrescine.down] (14%)
	[Both.down] (24%)
GO:0006952~defense response	[NH.up] (6%)
GO:0042420~dopamine catabolic process	[NH.up] (27%)
	[Putrescine.up] (27%)
	[Both.down] (36%)
GO:0071321~cellular response to cGMP	[Putrescine.down] (30%)
GO:0042493~response to drug	[Putrescine.down] (5%)
GO:0009712~catechol-containing compound metabolic process	[Putrescine.down] (100%)
GO:0050668~positive regulation of homocysteine metabolic process	[Putrescine.down] (67%)
GO:0045963~negative regulation of dopamine metabolic process	[Putrescine.down] (67%)
GO:0008203~cholesterol metabolic process	[Putrescine.down] (9%)
GO:0006979~response to oxidative stress	[Putrescine.down] (5%)
GO:0009395~phospholipid catabolic process	[Putrescine.down] (21%)
	[Both.down] (29%)
GO:0042744~hydrogen peroxide catabolic process	[Putrescine.down] (20%)
	[Both.down] (25%)
GO:0030163~protein catabolic process	[Putrescine.up] (6%)
**Signal transduction and phosphorylation**	
GO:0000160~phosphorelay signal transduction system	[Both.down] (26%)
GO:0007186~G-protein coupled receptor signaling pathway	[Both.down] (16%)
GO:0007166~cell surface receptor signaling pathway	[Both.down] (19%)
GO:0023014~signal transduction by protein phosphorylation	[Both.down] (19%)
GO:0090263~positive regulation of canonical Wnt signaling pathway	[Both.down] (17%)
GO:0018107~peptidyl-threonine phosphorylation	[Both.down] (15%)
GO:0006468~protein phosphorylation	[NH.up] (3%)
	[NH.down] (2%)
	[Putrescine.up] (3%)
	[Both.down] (8%)

GO:0007169~transmembrane receptor protein tyrosine kinase signaling pathway	[NH.up] (9%)
	[NH.down] (9%)
	[Putrescine.up] (11%)
	[Putrescine.down] (9%)
	[Both.down] (36%)
**Other**	
GO:0072384~organelle transport along microtubule	[Both.down] (75%)
GO:0007018~microtubule-based movement	[Putrescine.down] (6%)
	[Both.down] (15%)
GO:0030705~cytoskeleton-dependent intracellular transport	[Both.down] (20%)
GO:1901879~regulation of protein depolymerization	[NH.down] (100%)
GO:0042264~peptidyl-aspartic acid hydroxylation	[NH.down] (40%)
GO:0071555~cell wall organization	[Both.up] (5%)
GO:0072659~protein localization to plasma membrane	[Both.up] (13%)
GO:0072661~protein targeting to plasma membrane	[Both.up] (12%)
GO:0018401~peptidyl-proline hydroxylation to 4-hydroxy-L-proline	[Both.up] (11%)
GO:0002938~tRNA guanine ribose methylation	[Both.up] (50%)
GO:0001558~regulation of cell growth	[Both.up] (10%)
GO:0060285~microtubule-based flagellar cell motility (cilium-dependent cell motility)	[Both.down] (55%)
GO:0007155~cell adhesion	[Both.down] (13%)
GO:0051291~protein heterooligomerization	[Both.down] (19%)
GO:0070192~chromosome organization involved in meiotic cell cycle	[Both.down] (75%)
GO:0080156~mitochondrial mRNA modification	[Both.down] (75%)
GO:0003341~flagellar movement (cilium movement)	[Both.down] (19%)
GO:0007131~reciprocal meiotic recombination	[Both.down] (17%)
GO:0055091~phospholipid homeostasis	[Both.down] (43%)
GO:0000266~mitochondrial fission	[Both.down] (24%)
GO:0036159~inner dynein arm assembly	[Both.down] (22%)
GO:0043059~regulation of forward locomotion	[NH.up] (100%)
	[Putrescine.up] (100%)
GO:0032259~methylation	[NH.down] (4%)
	[Both.up] (5%)

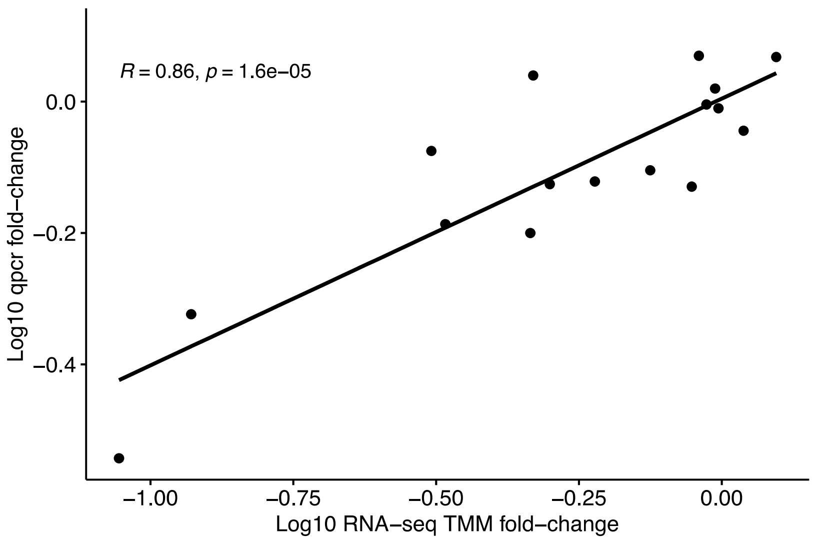

Figure S1. Correlation between the gene expression fold-change from RNA-seq and RT-qPCR methods.

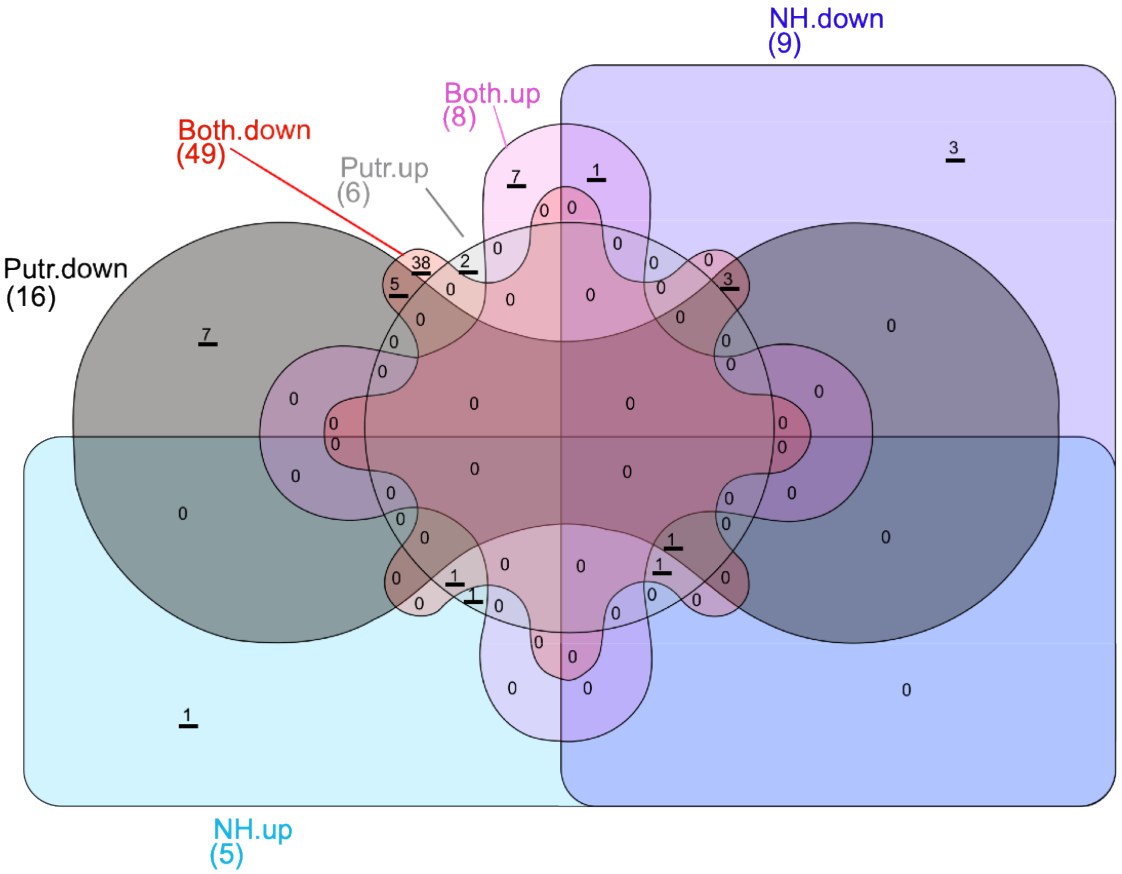

Figure S2. Venn diagram analysis. Venn diagram analysis of biological processes enriched by genes up- or down-regulated by ammonium (NH.up and NH.down), putrescine (Putr.up and Putr.down), or a combination of ammonium and putrescine (Both.up and Both.down). Non-zero numbers are underlined. The gene ontology enrichment analysis was conducted using DAVID (Huang et al., 2009b, 2009a); the Venn diagram analysis was conducted using InteractiVenn (Heberle et al., 2015).

References

Coyne, K. J. (2010). Nitrate reductase (NR1) sequence and expression in the harmful alga *Heterosigma akashiwo* (Raphidophyceae). J. Phycol. 46, 135–142. doi:10.1111/j.1529-8817.2009.00781.x.

Grabherr, M. G., Haas, B. J., Yassour, M., Levin, J. Z., Thompson, D. A., Amit, I., et al. (2011). Full-length transcriptome assembly from RNA-Seq data without a reference genome. Nat. Biotechnol. 29, 644–652. doi:10.1038/nbt.1883.

Haas, B. J., Papanicolaou, A., Yassour, M., Grabherr, M., Blood, P. D., Bowden, J., et al. (2013). *De novo* transcript sequence reconstruction from RNA-seq using the Trinity platform for reference generation and analysis. Nat. Protoc. 8, 1494–1512. doi:10.1038/nprot.2013.084.

Heberle, H., Meirelles, V. G., da Silva, F. R., Telles, G. P., and Minghim, R. (2015). InteractiVenn: A web-based tool for the analysis of sets through Venn diagrams. BMC Bioinformatics 16, 169. doi:10.1186/s12859-015-0611-3.

Huang, D. W., Sherman, B. T., and Lempicki, R. A. (2009a). Bioinformatics enrichment tools: Paths toward the comprehensive functional analysis of large gene lists. Nucleic Acids Res. 37, 1–13. doi:10.1093/nar/gkn923.

Huang, D. W., Sherman, B. T., and Lempicki, R. A. (2009b). Systematic and integrative analysis of large gene lists using DAVID bioinformatics resources. Nat. Protoc. 4, 44–57. doi:10.1038/nprot.2008.211.

Patro, R., Duggal, G., Love, M., Irizarry, R., and Kingsford, C. (2015). Salmon provides accurate, fast, and bias-aware transcript expression estimates using dual-phase inference. bioRxiv, 021592. doi:10.1101/021592.

R Core Team (2015). R: A language and environment for statistical computing. R Foundation for Statistical Computing, Vienna, Austria. URL https://www.R-project.org/.
